# Circulating Nucleic Acids in Colorectal Cancer: Diagnostic and Prognostic Value

**DOI:** 10.1155/2024/9943412

**Published:** 2024-02-13

**Authors:** Somayeh Igder, Mozhdeh Zamani, Shima Fakher, Morvarid Siri, Hassan Ashktorab, Negar Azarpira, Pooneh Mokarram

**Affiliations:** ^1^Department of Clinical Biochemistry, School of Medicine, Ahvaz Jundishapur University of Medical Sciences, Ahvaz, Iran; ^2^Autophagy Research Center, Shiraz University of Medical Sciences, Shiraz, Iran; ^3^Department of Biochemistry, School of Medicine, Shiraz University of Medical Sciences, Shiraz, Iran; ^4^Department of Medicine, Gastroenterology Division and Cancer Center, Howard University College of Medicine, Washington, DC, USA; ^5^Autophagy Research Center, Department of Biochemistry, Shiraz University of Medical Sciences, Shiraz, Iran

## Abstract

Colorectal cancer (CRC) is the third most prevalent cancer in the world and the fourth leading cause of cancer-related mortality. DNA (cfDNA/ctDNA) and RNA (cfRNA/ctRNA) in the blood are promising noninvasive biomarkers for molecular profiling, screening, diagnosis, treatment management, and prognosis of CRC. Technological advancements that enable precise detection of both genetic and epigenetic abnormalities, even in minute quantities in circulation, can overcome some of these challenges. This review focuses on testing for circulating nucleic acids in the circulation as a noninvasive method for CRC detection, monitoring, detection of minimal residual disease, and patient management. In addition, the benefits and drawbacks of various diagnostic techniques and associated bioinformatics tools have been detailed.

## 1. Introduction

Colorectal cancer (CRC) is the third most prevalent cancer in men and women and the fourth cause of cancer-related mortality [1]. About one in five CRC patients present late-stage disease when diagnosed [2]. Since CRC has a poor prognosis and a high mortality rate at advanced stages, early detection of the malignancy, especially with noninvasive methods, has gained momentum [3]. The American Cancer Society has recommended CRC screening for average-risk individuals consisting of select stool-based tests or visualization examinations of the colon and rectum [4]. Colonoscopy and the evaluation of the biopsies by histopathology are the golden standards for CRC diagnosis. These methods are invasive and time consuming, which is why scientists have turned to less invasive techniques such as those that are stool based, like the use of stools in Cologuard testing, where few host genetic and epigenetic markers are tested to establish CRC risk scores. Randomized controlled trials have proven that the fecal occult blood test can detect CRC and significantly lower the rate of death from the disease [5]. A fecal-occult blood test (FOBT) is a noninvasive test that detects hidden (occult) blood in the stool. Such blood may come from anywhere along the digestive tract, and for that reason, additional types of diagnostic tests (colonoscopy, flexible sigmoidoscopy, and double-contrast barium enema) may be ordered. Blood in the stool may be the only symptom of early cancer [6]. Carcinoembryonic antigen (CEA) is the most common CRC marker. It is usually released by tumors in the digestive tract. CEA has reliable sensitivity and specificity for screening CRC and is a powerful biomarker for assessing the CRC prognosis. There is not a single serum tumor marker that can accurately diagnose CRC, so we have to pick and combine a group of tumor markers or do more tests to get a more accurate result [7]. Recent improvements in finding circulating tumor markers in blood have made blood-based screening more popular among both patients and doctors. This is because it can find potential cancers anywhere in the body as long as tumor cells are shed into the bloodstream. However, the sensitivity and specificity issues of some serum-based methods delayed the early detection of CRC [8]. Fragmented DNA within the circulation, urine, and other body fluids produced by cells is referred to as cfDNA. It exists at low concentrations in fragments shorter than 200 base pairs that are not attached to cells or organelles [9]. The concentrations of cfDNA, which originates from cells' apoptosis or necrosis, are less than 100 ng/ml in the circulation of healthy individuals, while in cancer patients, the level of cfDNA and also the tumor-derived fraction of cfDNA or ctDNA are much higher [10]. Among the cancerous genome, ctDNA is composed of short fragments (around 150–200 base pairs) that are secreted by cancer cells entering the blood circulation with a transient half-life lower than a few hours, which indicates that it can reveal driving cancer traits. Significantly, the clinical efficacy of cell-arising DNA or RNA corresponds to the conservation of epigenetic information and the potential assessment of cancer-associated mutations in peripheral blood, stool, and urine as a valuable diagnostic utility for CRC. Recent research suggests that ctDNA comes from cancer cells that have died (apoptotic or necrotic), cancer cells that are circulating, and cancer cells that are changing. Using circulating cell-free (tumor) RNA (cfRNA/ctRNA) analysis along with ctDNA could help figure out the molecular structure and find many differences linked to the tumor [11, 12]. Looking into the amount of cfDNA in blood samples shows that levels range from 59 ng/ml in healthy people to 156 ng/ml or higher in people whose colon cancer has spread [10]. Here, we will emphasize that cfDNA/ctDNA and cfDNA/ctDNA and cfRNA/ctRNA are distinguished in the liquid biopsy of CRC cases with different stages that were first introduced in the diagnosis of prostate cancer, and now they may be of interest in the diagnosis of CRC. Accordingly, we draft this review by supplying a summary of the foremost techniques applied to evaluate genetic and epigenetic abbreviations in these circulating tumor nucleic acids (CtNAs) as an approvable implementation for the diagnosis and management of CRC.

## 2. Circulating Tumor Cells (CTCs)

### 2.1. Strategies for Isolating and Characterizing

CTCs are tumor cells that have infiltrated the bloodstream after separating from tumor tissue. In order for patients to develop distant metastases, it is necessary for them to generate CTCs. CTC production is an essential requirement for patients who are developing distant metastases [13]. In the context of CTCs, the optimal technique for separation, enrichment, and detection must strictly adhere to stringent criteria encompassing both sensitivity and specificity. Positive collection analyses, including the Cell Search system (ferro fluid nanoparticles-based EpCAM detection), AdnaTest (identification of expression patterns of assembled antibody-coated beads by reverse transcription-polymerase chain reaction (RT-qPCR)), magnetic-activated cell sorting (MACS) (based magnetic nanoparticles coated by antibodies), and microfluidic-based positive enrichment technologies (through tetramerization), are utilized to isolate these cells [14]. The EasySep system (magnetic nanoparticles and antibodies targeting CD45 and CD61 cells) and quadrupole magnetic separation (QMS, assessment of immunomagnetically labeled cells) are two major groups of immunoaffinity-based negative purification techniques that typically eliminate white blood cells (WBCs) from blood specimens by targeting antigens that are not carried by CTCs. Biophysical CTC extractions based on their distinct firmness, size density, and electrical burden relative to background cells are supplementary purification methods. Immunohistochemistry is utilized to distinguish and isolate CTCs, while DNA replication and variation analysis are simultaneously possible. Thus, genetic translocations or amplifications within CTCs were additionally identified via FISH assays [15, 16]. In situ hybridization (ISH) or sequencing can be utilized to analyze the RNA; the latter identifies a unique RNA sequence in cancer cells that are in circulation. A CTC capture system is Hydro-Seq, a microfluidic device that Cheng et al. [17] recently developed. It successfully isolates ultrapure CTCs from patient blood samples with high accuracy while remaining unaffected by red blood cells and WBCs. Clinicians are empowered to administer efficacious treatment to patients by means of hydro-seq's capacity for a comprehensive, high-throughput analysis of CTCs [17]. A recently developed engineering technique involves the fabrication of biomimetic nanoparticles, which are composed of synthetic nanomaterials blended with natural biomaterials, including platelet, leukocyte, and RBC membranes. On a microfluidic substrate, a fluidic and multivalently engineered nanointerface with an aptameric-functionalized leukocyte membrane nanovesicle has been characterized [18]. Incorporating anti-EpCAM-modified cell membranes into platelet–leukocyte hybrid membrane-coated magnetic nanoparticles enabled the efficient isolation of CTCs. When nanoparticles are mixed with cell membranes, the chance of them sticking to and getting rid of other particles is greatly reduced. In addition, interfacial collision-induced cell injury can be mitigated by positioning a layer of flexible, delicate nanovesicles between the cell and the capture substrate [18].

### 2.2. Technical Difficulties Associated with CTC Separation and Molecular Quantification

CTCs are anticipated to become novel biomarkers for the diagnosis and evaluation of cancer treatments. Molecular insights for therapeutic monitoring in clinical trials and early cancer diagnosis may be unveiled through the expanding field of single-cell analysis of CTCs. Several factors, such as CTC susceptibility, heterogeneous biology, and low concentrations, can change the outcome of a single CTC analysis [19].

In order to achieve high throughput, purity, selectivity, and viability of CTC enrichment, an isolation platform must be chosen; immunocapture platforms generally provide a more pure enrichment [20]. However, it is possible for several of these methods to fail to detect certain CTCs due to their minimal or nonexistent epithelial marker expression, which could specifically exclude the most invasive tumor cells. Specific types of cancer cells have the ability to transition partially into a mesenchymal state, which can hinder the effectiveness of marker-based approaches that rely on the time required for ligand acquisition and cell binding. Despite the fact that physical-principle-based separation techniques provide high throughput and efficient analysis, the heterogeneous biology of CTCs continues to present obstacles [21]. By way of illustration, the loss of metastatic tumor cells and an escalation in the proportion of blood cell contamination may be influenced by the physical properties of CTCs and blood cells. Physical-principles-based separations can have unintended consequences for cell viability, influenced by elements like pressure, tension, an electric field, or conductive media. Further investigation is required to determine whether or not concentrations of CTCs contribute to metastasis. Proteomic and genomic heterogeneity is considerable between solitary and clustered CTCs [22]. CTC cluster research is significantly hampered by the fact that the majority of existing isolation technologies are devised to capture individual cells rather than clustered CTCs. Because their respective benefits can be combined and platform-specific obstacles can be bypassed, platforms that incorporate numerous capture principles have the potential to be extraordinarily beneficial. For clinical diagnosis, the aforementioned considerations must be incorporated into the design of procedures that achieve complete CTC enrichment with high throughput, efficiency, purity, and viability. Analysis of a single CTC necessitates substantial expansion in order to provide a comprehensive view [22]. For single-cell analysis, which will be used to determine the efficacy of the enrichment method, the integrity of the isolated cells is invaluable. Viability is the paramount consideration, particularly in the context of drug efficacy evaluations at the level of a single CTC and culture experiments. Accordingly, the separation procedure must ensure cell viability and purity of CTCs for genome and protein profiling, allowing for adequate materials for characterizing heterogeneity through large-scale single-cell data [23]. Insufficient enrichment purity can introduce enigmas, making single-cell data analysis more challenging [14]. The difficulties associated with molecular quantification and semiautomated CTC separation are reviewed in Table 1.

## 3. Circulating Nucleic Acids

Extracellular nucleic acids, such as DNA and RNA, circulate throughout the body after normal, primary malignant, and metastatic cancer cells secrete them circulating nucleic acids (CNAs) in plasma and serum, present in both benign and malignant conditions, significantly impact minimally invasive diagnostic and prognostic applications [31]. Cell-free DNA (cfDNA), also known as circulating tumor DNA (ctDNA), can be detected in the plasma of individuals diagnosed with cancer. ctDNA has been employed in various aspects of cancer research ever since the initial documentation of identical DNA mutations in plasma and tumors, including diagnosis, detection, prognosis, treatment selection, and surveillance. Cancer patients can be distinguished from healthy individuals based on the quantity and quality of their circulating cfDNA. In general, cancer patients have higher cfDNA concentrations than healthy individuals, and these concentrations appear to rise with metastasis and disease progression [32]. An increased discharge of genetic material from tumor cells or impaired phagocyte clearance could account for the elevated cfDNA levels observed in cancer patients. However, their immediate application in cancer diagnosis may be impeded by the presence of elevated cfDNA levels in conditions such as trauma, exercise, and surgery [33]. Following circulating tumor cell apoptosis in the blood and urine of patients diagnosed with various malignancies, exosome-like particles have been found to be enriched with tumor-derived circulating RNAs (ctRNA), including mRNAs and especially small RNAs (miRNAs and long noncoding RNAs). Liquid biopsies are increasingly being considered as a viable transition to clinical practice due to their less invasive, simpler, quicker, and less expensive access to body fluids [34].

## 4. cfDNA Detection

Tables 2 and 3 show a summary of the different standard tests that were used to measure the amount of cfDNA-associated key biomarkers in CRC samples. The cfDNA is made up of DNA fragments from different sources that are all very different sizes, and the ctDNA makes up less than 1% quantifying such low volumes is problematic, particularly with the low disease burden at the initial stages [80]. Here, two workable strategies can effectively be followed. The first strategy involves noninvasive targeted identification of specific tumor mutations already detected in the primary tumor, especially in postsurgical monitoring using cfDNA. However, this mutation detection rate is limited to low-frequency mutated genes (referred to as low variant allele frequency (VAF)) at approximately 0.01%, along with a higher bar for specificity and faster assay times. The second approach emerged as an undirected strategy, which emphasizes genome or whole exome deep sequencing tumor-correlated copy number alterations analysis or somatic mutations without matched prior interrogating tumor analysis. To achieve this, accurate methodologies are implemented: real-time quantitative PCR (qPCR), NGS, digital PCR, the BEAMing procedure, and mass spectrometry. The traditional assay for cfDNA quantification was real-time qPCR, which determined the amplification concentration variation along with missense substitutions and insertion/deletion events with higher than 10% VAF [88]. However, present high-throughput analytical technologies are associated with more precise detection rates through a limited extension step at the 3′ end probe and high-frequency mutant allele-specific proliferation. Technologies based on the NGS ultradeep the next-generation panel covers diverse procedures planned to filter out low VAF variants with great confidence based on the hotspot regions, resulting in an acceptable level of analytical sensitivity of about 100% and a desired specificity of nearly 80% [89]. Up to this point, the robust and high sensitivity of around 98% for scanning and pinpointing novel point mutations is supported by the tagged amplicon deep sequencing (TamSeq), safe-sequencing system (Safe-SeqS), cancer personalized profiling by deep sequencing (CAPP-Seq), bias-corrected targeted NGS, and multiplex PCR NGS methods. Bias-adjusted targeted NGS provides multivalued markers involving samples and divergent sequence tags physically linked to oligonucleotide capture probes, which are subsequently proliferated with elevated significance [90]. Here, approved commercial protocols for WGA of the plasma-circulating genome are Sigma-modified. WGA2 or Sigma-modified WGA4 methods, although no independent validation has been disseminated. Unpublished results showed that cfDNA fragments in the 150-bp size range are also small for WGA quantification. To our knowledge, more detailed scientific evidence of the technical procedures and suggestions in the course of sample processing for cfDNA tests was defined by the European consortium CANCER-ID (https://www.imi.europa.eu/projects-results/project-factsheets/cancer-id) in the framework of the Innovative Medicines Initiative (IMI). In addition, the European SPIDIA project (http://www.spidia.eu/) has also addressed official CEN/technical characteristics documents relevant to standards on the processing, preserving, and attestations of venous whole blood samples in preparation for cfDNA during the preanalytical phase [88]. Digital PCR (dPCR) corresponds to a reduced overall time and cost per run when compared to NGS but does not permit a parallel review of the majority of actionable genomic deviations. Techniques called beaming, which rely on beads, emulsion, amplification, magnetics, and droplet digital PCR (ddPCR), are the two main technologies for single-molecule counting. Alternatively, ddPCR can be done on a water–oil emulsion platform where DNA samples are spread out over thousands of microemulsions in both mutant and WT reactions. By using fluorescently labeled probes that are specific to a site, target amplicons can be analyzed using flow cytometry. Also, ddPCR seems to be a lot more profitable than regular digital PCR, which involves handling serial dilutions of intact DNA into separate wells for detection, which can be hard at times. Liquid biopsy is considered a surrogate validated noninvasive method for dynamical monitoring of tumor-derived circulating mutant alleles in standard clinical applications for advanced CRC cases [91]. In contrast to tissue biopsy approaches, it reflects tumoral heterogeneity, clonal development, and diffuse patterns [92]. In view of the high analytical precision, it is well-established that dPCR technologies naturally merge more sensitivity than conventional qPCR techniques for quantifying rare somatic mutations amongst wild-type genomic backgrounds in cfDNA as well as KRAS-mutated ctRNA [93]. The fully automated IdyllaTM RAS mutation method is a qPCR test that is performed in two separate consecutive runs with a reported analytical sensitivity rate of ≤1% for tumors harboring hotspot KRAS point mutations in exons 2 and 3 and ≤5% for CRC-associated rare mutations centered on KRAS exon 4 [84, 93]. While the OncoBEAMTM RAS CRC, known as a modified dPCR, diagnostic molecular testing based on BEAMing technology analyzed KRAS and NRAS oncogenic mutations synchronously with ultrasensitive analytical and diagnostic accuracy down to 0.02% mutant allelic fraction (MAF) [84, 94]. In the BEAMing procedure, each allele's particular polymerization was assessed on magnetic beads in emulsion PCR through hybridization with wild-type or mutant sequence-targeted fluorigenic probes. Two interesting things about the OncoBEAM RAS CRC technology are that it can give more accurate results for plasma KRAS mutation assaying in patients with mCRC than the Idylla system [67] and that it can do a wider range of quantitative tests [95]. In this setting, the OncoBEAM TMRAS CRC plasma test can be incorporated into the early histological report to enable careful prediction of targeted therapy responses and holistic genetic mutational trialing for new histologically authenticated mCRC [96]. The lower threshold of mutational allelic detection in the OncoBEAMTMRAS CRC experiment with a minimum of 0.03% is also noteworthy as it lowers the number of plasma-derived cfDNA templates [67]. This screening platform could be so successful regarding kinetical assaying of the mutated haploid GE quantities during cancer patient therapy since the possibility of longitudinal checking of sequential tissue biospecimens is not practical [67]. The notion of MAF as an establishing prognostic instrument was approved by the investigation, which found that mCRC patients with an increased KRAS mutation fraction (higher or equal to 1% MAF sensitivity) tended to have shorter progression-free survival (PFS) rates, especially compared with patients with tumors bearing KRAS mutations below 1% MAF [67]. Furthermore, it has also been concluded that fast-progressing cases carried significantly higher levels of MAF than slow-progressing cases. Consequently, this evidence can be easily incorporated to notify the clinicians so they can provide an information-based estimate of survival and recurrence of malignant neoplasms in high-risk CRC patients for better management and follow-up [93].

## 5. ctRNA Detection

Mass spectrometry is applicable for surpassing obstacles in PCR multiplexing by designing specific nanotags that are subsequently attached to wanted hotspot areas and emit through laser excitation or biotin markers, which can detect about 40 targets with 5 ng of starting material per reaction [97]. High-throughput RNA sequencing of short noncoding RNAs under 30 nt long, like miRNAs, piRNAs, and endosiRNAs, can successfully be procured by direct ligation with adapters without the need for further RNA handling ahead of the ligations of the first copies (thanks to the 5′-terminal phosphate and 3′ hydroxyl units of miRNAs). Nevertheless, this assay presents considerable bias resulting from the impact of sequence on ligation. The circularization of the single-stranded cDNA with DNA ligase and their amplification using PCR, especially for 5′-adapter ligation vs. 3′-adapter ligation, can mitigate biases. In addition, incorporating degenerate random nucleotides at the ligation ends of adapters and applying a particular electrophoresis separation or locking of the 3′-adapter by inserting the RT primer can prevent contaminant PCR products devoid of each insert from ligating with the 5′-adapter (Table 2) [54].

## 6. Consideration for Optimizing Preanalytical Procedures

### 6.1. Preanalytical Laboratory Factors

The analytical sensitivity and clinical efficacy, as well as the diagnostic validity of cfDNA/RNA-based assays, will require the determination and management of different factors, such as critical preanalytical laboratory factors, and bioinformatics data processing [98]. Investigating and optimizing the effect of the type of blood collection tube is the first critical preanalytical laboratory factor. The current application of serum vs. plasma has been discussed [54]. There are two significant obstacles when handling biospecimens (serum or plasma) to isolate sufficient amounts of cfDNA prior to downstream quantification analysis. The first is a low concentration of extracted cfDNA or ctDNA, determined as either ng/ml of plasma or up to 1,000 copies per milliliter of blood. The second is that the resulting cfDNA, collected in a serum blood collection tube (BCT), is often contaminated by large genomic DNA segments released from WBC, which mandates proper WBC effect removal to provide better specificity for the subsequent processes [26, 27]. The isolated total cfDNA levels constantly generate five-to-eight-fold greater yields in fresh serum compared to those in plasma. However, serum is more variable and harder to manage due to the coagulation procedure and is not suggested as the starting material for dedicated cfDNA extraction kits. Thus, anticoagulated whole blood is preferred for cfDNA-based genetic studies and ctDNA analysis because plasma specimens are a better source than serum. Subsequently, EDTA has been recommended as a better anticoagulant than citrate or heparin (as an inhibitor of PCR). EDTA maintains cfDNA integrity in plasma through the inhibition of DNase activity, so the use of EDTA 2K tubes followed by two-step centrifugation has been considered the standard approach for genetic analysis [99].

Moreover, it has already been reported that high-MW spiked DNA gathered into serum BCT was not restored or identified electrophoretically in the isolated serum cfDNA, unlike cfDNA BCT plasma. It emerged that such high MW DNA should have been captured in the clot within the coagulation process. Another report by Warton et al. [100] Parpart-Li et al. [101] showed a shift to considerable extra whole genome equivalents (GEs), but considerably lower circulating mutant allele frequencies in serum when using EDTA than plasma samples from tumor subjects. The size profile of the total cfDNA fragments extracted from serum varied from 150 to 2,000 bp, while plasma samples corresponded to a unique dominant peak of 150 bp. The most sensitive platelet-producing protocol has been demonstrated to unbind major numbers of platelet microparticles and miRNA containing the maximum cfRNA output from stock plasma specimens through a single freeze/thaw cycle, unlike platelet-poor plasma (PPP), which required two processes of centrifugal force before freezing. Other preanalytical parameters involve remainder cells and microparticles impurities such as CTCs, minor (cancer-sourced) EVs, and cfDNA (<3%), major (cancer-sourced) extracellular vehicles (EVs; 22%), and red blood cells (involved in plenty of RNAs) that could negatively affect the real power of cfRNA measuring. The storage of whole blood in sodium EDTA tubes in parallel at 4°C revealed no cirDNA concentration alteration for up to 1 day. Whether the anticoagulated blood must be stabilized or not and which type of chemical stabilizer should be used is still being investigated [28, 29]. Cell lysis also appears in EDTA tubes over the long-term preservation time of collected blood, mainly at room temperature (RT). Subsequent WBC lysis, cellular genomic DNA debris, and DNases are released. DNases may degrade the cfDNA, despite the fact that EDTA can inhibit, to a determined extent, endogenic DNases [30]. Blood cell lysis is efficiently prevented by commercially developed stabilizers used in Streck, Cell Save, Roche, Norgen Biotek, or PAXgene blood cfDNA/ctDNA tubes. Similar DNA yields and proficiency of cfDNA from Streck BCT, Roche, and PAXgene cfDNA tubes, with accurate qPCR detection of 0.5 ng spiked mutant DNA, are detected after 7 days of RT blood incubation in all tubes. BCT tubes seemed to maintain blood cell integrity and whiten out any increased DNA concentration for up to 7 days (for ctDNA extraction, the range was from 48 hr to 5 days at RT) following blood collection. Furthermore, BCT tubes support the actual cirDNA concentration as a well-known tool for blood conservation and stabilization in ideal cirDNA quantification assays. Some cell stabilizers that contain formaldehyde cross-linking reagents, such as Streck, contribute to the methylation pattern of cfDNA through the induction of cfDNA deamination, which introduces variations in cfDNA methylation quantification. On the contrary, alternative PAXgene cfDNA tubes enable the unchanged data detection of sequence-specific methylation cfDNA status and are consequently appropriate for target downstream cfDNA methylation measurement. Recently, a study by Holmes et al. [102] showed that no significant discrepancy in relation to the background error rate between cfDNA purified from Streck BCT preservative tubes and paired standard EDTA tubes was obtained for all amplicons in the Tagged Amplicon deep sequencing method (Tam-Seq). In addition, these cell preservative tubes were ideal for the purpose of the cfDNA extraction in the scope of tumor-derived subchromosomal copy number variation (CNV). The selection of temperature storage and sampling time for serum or plasma preparation from blood cells is the second most important variable in the preanalytical phase. For example, a broad range of biospecimen clinical research surveys reveal that EDTA blood processing should be applied in a maximum of 3–6 hr following blood draws if samples are kept at RT. Even though blood samples are stored for 8 or 24 hr in commercial EDTA tubes in a refrigerator at 4°C, the delay in purification can be avoided using the QIAamp MinElute cfDNA Kit. Blood collection Streck BCT and PAXgene tubes with cell-stabilizing reagents generally seem to be superior for cfDNA yield at 25°C up to 7 days' storage, based on manufacturer claims, compared to the lower stability of the cfDNA collection tube from Roche Diagnostics GmBH. Regarding the centrifugation conditions, such as a double-spin plasma preparation protocol followed by a second high-speed centrifugation step at 16,000 *g* (3,000–16,000 *g*) for 10 min, this influences the presence of high-quality cfDNA purification [28, 102].

The third critical factor leading to preanalytical biospecimen handling is the type of cfDNA purification kit. Tables 2 and 3 list the broadly utilized extraction kits and techniques. A parallel review between several different kinds of cfDNA extraction kits and procedures reported the highest purified cfDNA outputs with the Norgen Kits. However, these various extraction tools may produce different fractions of plasma cfDNA molecules with a wide size variety based on the DNA-capturing capacity of the beads or silica gel membranes used during the purification treatment [103]. Additionally, it should be considered that differentially high-molecular-weight DNA originating from necrotic malignant tumor cells could be retained in the extracted combined cfDNA eluate over a variety of existing kits, compatible with other extraction approaches. The molecular mechanisms underlying nucleosome release into circulation are associated with apoptosis-dependent cell death upon targeted therapies like tyrosine kinase inhibitors. This information must be taken into consideration when tracking mutations in cfDNA for therapeutic drug monitoring [104].

More recently, comparative studies between six various cfDNA extraction platforms based on magnetic-bead technology showed that the high-throughput cell-free circulating DNA isolation kits from input plasma volumes of 4 ml by QIAGEN and Norgen Biotek's companies improved DNA fragment recovery with a wide length range (50–808 bp). While the Applied Biosystems isolation kit (wide sample volume inputs range from 500 *µ*L to 10 ml of plasma or serum) leads to no carryover of shorter size fragments than 50 bp relative to other reliable separation strategies associated with PerkinElmer kits that delivered significantly efficient retrieval of DNA molecules in the size range of 75–300 bp from 0.5 to 1.5 ml of plasma or serum samples [28, 105, 106], for systemic cfDNA methylation profiles, a potential adapted MethylMiner (Invitrogen) method for the extraction of plasma-derived methylated cfDNA variants has been described. Another considerable preanalytical variable is deterioration due to long-term cfDNA storage at −80°C. The incorporation of bisulfite conversion treatment is also an important preanalytical factor. Currently, many kits are employed for methylated DNA molecular pattern studies, but one product that is most widely fitted for the bisulfite conversion process of plasma-based cfDNA is the InnuCONVERT Bisulfite Body Fluids Kit (Analytik Jena AG), which performs with a maximum 3 ml plasma input volume [28, 88]. The Epitect (Qiagen) Kit is best suited for efficient bisulfite conversion performance on purified cfDNA with “a limited quantity” of fragmented DNA. Methylation-on-beads (MOB) is a new integrated sample extraction and processing technique that facilitates cfDNA isolation and bisulfite treatment for up to 2 ml of plasma or serum and guarantees great recovery and assay sensitivity of the CpG methylation status analysis.

Systemic chronic stress associated with physical or mental events and acute stress conditions such as intense exercise followed by muscle injury and repair responses trigger long-term release of DNA, together with a subsequent significant increase in cfDNA levels during cellular apoptosis or necrosis and dynamic variations in cfDNA methylation profiling. Additionally, acute plasma viral reactivation ratios corresponding to HIV, hepatitis B, and Epstein–Barr infections result in a high percentage of cfDNA concentrations because of the high cell-free viral DNA load. The amount of tumor-of-origin cfDNA is likely further influenced by clinical variables, including clinical stage, histopathological grading, primary, local, or distant recurrence pattern, and response rate to targeted therapy, which reveals more applications of cfDNA tests in clinical diagnostics and prognosis. Similarly, a major potential association between circadian rhythmicity and a more elevated cfDNA amount at midday was recognized in patients with stages I–III CRC compared with cases with stage IV [35]. The genotoxic effects of environmental agents like pesticides on the increase in cfDNA concentration were described previously, as exposed women harbored a higher cfDNA level than exposed men [107]. Age can also be an especially significant predictor when one surveys relative input amounts of cfDNA at basic genomic sites, in particular, transcription initiation and termination regions, or organ-specific and cfDNA-correlated methylation footprints [27, 28, 88]. Multiple studies have demonstrated conclusively that genomic and environmental factors modulate an individual's cfDNA level, making it highly variable within the healthy population. Our study suggests that the diagnostic sensitivity of cfDNA evaluation as a noninvasive biomarker could be enhanced if the individual's cfDNA level is known prior to the onset of disease or cancer. Plasma cfDNA levels could therefore serve as a sensitive, noninvasive biomarker for the diagnosis and prognosis of numerous diseases, particularly malignancies, if further validation in larger cohorts is achieved. On the other hand, neither alone nor in isolation, CTCs can enhance cancer diagnostic and prognostic applications. For instance, concluded that the combined analysis of cfDNA and CTCs provides additional information for identifying patients with a poor prognosis, as the sensitivity to detect relapses increased from 79% to 90% within 2 years. A second study utilizing NGS assays based on PCR demonstrates the usefulness of this combined analysis method for cancer diagnosis. For example, concluded that the combined analysis of cfDNA and CTCs provides some extra information to detect patients with a worse prognosis, as the sensitivity to detect relapses increased from 79% to 90% within 2 years. Another study using a PCR-based NGS assay proves this combined analysis method is useful in cancer diagnosis. The high integrity of miRNAs in circulation blood contributes to establishing miRNA expression patterns and takes advantage of its potential for reliable spotting over combined assay-based TRIzol material isolation procedures with spin columns, marketing accessible kits (Table 4), and immunomagnetic beads coated by capture antibodies for exosomal miRNAs. Although the coextraction TRIzol protocol for selective circulating miRNA isolation has the possibility of cross-reactivity with DNA, lipids, or proteins and cell-derived microparticles-like platelets or erythrocytes, besides phenolic contaminants [12, 112].

### 6.2. Bioinformatics Data Processing

Bioinformatics data processing is an important part of cfDNA oncological research because it helps find patterns of point mutations, insertions and deletions, genomic CNVs, and abnormal DNA methylation profiles that are unique to each patient. In addition to the low-cost WGS-based CNV method, depth of coverage methods like QDNA-seq, WisecondorX, BIC-seq2, and the CNV kit are being used more and more to look at the landscape of genomic copy number information from the sequence depth. It is also possible to describe CNVs and chromosomal changes using the assembly-based, split-read, and read-pair methods. Before the sequencing alignment process, Y pseudo-autosomal regions and genomic regions with low map ability need to be removed so that the short reads can be mapped to a single genomic position instead of several likely positions. This is especially important for reference-free methods. So, the GEM (GEnome Multitool) map ability algorithm is a useful tool for improving single genomic map ability information and filtering of genomic regions with too many unstructured anomalous reads mapping in very genome-wide inconsistent regions, like different haplotypes overexpressed on chromosome 19 or problematic centromere, telomere, and satellite repeats [37, 53, 113, 114].

Before moving on to more in-depth analysis, these variable regions were found using a data-driven method with the ENCODE and mod ENCODE consortia and the detection QDNAseq algorithm, which can be downloaded for free from https://sites.google.com/site/anshulkundaje/projects/blacklists and https://bioconductor.org/packages/QDNAseq/, respectively. It has been suggested that subsections harbor individual DNA methylation epi-signatures and abnormal large-scale and locus-specific methylation patterns from each tissue type that can be used to differentiate between cancer and normal cells. Thus, complete plasma cfDNA genomic methylation profiling could be a potentially promising tool to identify tumor-type-specific tissue using cfDNA. Subsequently, first proved the practicability of leveraging large-scale genomic methylation expression databases from the Cancer Genome Atlas (TCGA) and Gene Expression Omnibus archives to distinguish distinct CpG dinucleotides for motif unmethylation and methylation status across a tissue of interest versus other tissues. By using methylation signature data that are tumor tissue-specific and appropriate filtration algorithms, a widely well-defined algorithm-based process to enhance the standard signal from a complex of signal origins might be employed to scheme reference sources of tissue using cfDNA [37, 53]. As shown recently, a probabilistic modeling method termed Cancer Locator has been developed to synchronously deduce the individual type of cancer based on cfDNA and the tissue proportion of ctDNA-derived fragments, mainly for samples with low to moderate ctDNA library yield directly from whole-genome DNA methylation data. Indeed, by comparing multidimension Infinium HumanMethylation450 microarray data from the TCGA project between matched normal and cancer specimens, Cancer Locator proved to be a useful tool for feature input data focusing on local clustering of CpG sites in high dimension with large interindividual DNA methylation across-tissue variation among different tumor types and normal groups [37, 114]. Millions of short transcripts are the primary point of RNA-seq computation. Initially, the monitoring control focuses on short-read sequencing using various designed databases like PRINSEQ and FastQC, as well as results handling to filter transcripts with low-quality bases, adapter sequences, and other foreign sequences from the raw sequencing output using Cutadapt and Trimmomatic tools and following raw reads mapped or aligned to a citation genome or transcriptome through TopHat2, STAR, GSNAP, OSA, and Map Splice algorithms. The mapped reads for individual tests are then evaluated on gene (involving RSEM, Cufflinks, IsoEM, feature counts, and HTSeq), sequence-based approaches (such as RSEM), or union-exon-based counting methods (like feature counts) to analyze the affluence of each category according to the experimental objective. The aforementioned statistical patterns (DESeq, edgeR, GENECounter, NOISeq, NBPSeq, and Cuffdiff2 approaches) then examine the RNA-seq count data to identify differentially expressed genes. Finally, pathway or network-level assays count on annotation websites including Gene Ontology (GO), Kyoto Encyclopedia of Genes and Genomes (KEGG) pathways, Database for Annotation, Visualization, and Integrated Discovery (DAVID), and additional commercial information systems, in particular, Ingenuity Pathway Analysis, to acquire biological imagination across systems biology strategies [115]. Also, it has already been said that high-MW spiked DNA collected in serum BCT could not be restored or identified electrophoretically in the separated serum cfDNA, but it could be in cfDNA BCT plasma. It emerged that such high MW DNA should have been captured in the clot within the coagulation process. Another study by Parpart-Li et al. [101] and Warton et al. [100] found a change to a lot more whole genome equivalents (GEs), but a lot fewer circulating mutant allele frequencies in serum when EDTA was used compared to plasma samples from people with tumors. The size range of the cfDNA fragments taken from serum was from 150 to 2,000 bp, but there was only one main peak at 150 bp for plasma samples. The platelet-producing protocol that has been shown to be the most sensitive can release most of the platelet microparticles and miRNA with the highest cfRNA output from stock plasma samples through a single freeze/thaw cycle. This is in contrast to PPP, which needed two cycles of centrifugal force before freezing. On the other hand, only negative levels of cfRNA were found in cell-free DNA BCT Streck tubes (La Vista, NE). This is likely because the cfRNA was not properly swollen, even after centrifuging. Other things that need to be looked at before analysis starts include leftover cells and impurities like CTCs, small (cancer-sourced) EVs, cfDNA (<3%), large (cancer-sourced) extracellular vehicles (EVs; 22%), and red blood cells (which contain many RNAs) that could make cfRNA measuring less accurate. The storage of whole blood in sodium EDTA tubes in parallel at 4°C revealed no cirDNA concentration alteration for up to 1 day. Whether the anticoagulated blood must be stabilized or not and which type of chemical stabilizer should be used is still being investigated [28, 29]. Cell lysis also appears in EDTA tubes over the long-term preservation time of collected blood, mainly at RT. Subsequent WBC lysis, cellular genomic DNA debris, and DNases are released. DNases may degrade the cfDNA, despite the fact that EDTA can inhibit, to a determined extent, endogenic DNases [28, 30]. Blood cell lysis is efficiently prevented by commercially developed stabilizers used in Streck, Cell Save, Roche, Norgen Biotek, or PAXgene blood cfDNA/ctDNA tubes. Similar DNA yields and proficiency of cfDNA from Streck BCT, Roche, and PAXgene cfDNA tubes, with accurate qPCR detection of 0.5 ng spiked mutant DNA, are detected after 7 days of RT blood incubation in all tubes. BCT tubes seemed to maintain blood cell integrity and whiten out any increased DNA concentration for up to 7 days (for ctDNA extraction, the range was from 48 hr to 5 days at RT) following blood collection. Also, BCT tubes help with the concentration of cirDNA and are a well-known way to keep blood safe and stable during ideal cirDNA quantification assays. There are some cell stabilizers, like Streck, that contain formaldehyde cross-linking reagents that change the methylation pattern of cfDNA by starting cfDNA deamination. This causes changes in the quantification of cfDNA methylation. On the other hand, different PAXgene cfDNA tubes allow for the same-level detection of cfDNA sequence-specific methylation status and are therefore suitable for measuring downstream cfDNA methylation. A study by Hrdlickova et al. recently found that there was no big difference in the background error rate between cfDNA purified from Streck BCT preservative tubes and paired standard EDTA tubes for all amplicons in the Tagged Amplicon deep sequencing method (Tam-Seq) [34, 89]. In addition, these cell preservative tubes were ideal for the purpose of the cfDNA extraction in the scope of tumor-derived subchromosomal CNV. The selection of temperature storage and sampling time for serum or plasma preparation from blood cells is the second-most important variable in the preanalytical phase. For example, a broad range of biospecimen clinical research surveys reveal that EDTA blood processing should be applied in a maximum of 3–6 hr following blood draws if samples are kept at RT. Even though blood samples are stored for 8 or 24 hr in commercial EDTA tubes in a refrigerator at 4°C, the delay in purification can be avoided using the QIAamp MinElute cfDNA Kit. Blood collection Streck BCT and PAXgene tubes with cell-stabilizing reagents generally seem to be superior for cfDNA yield at 25°C up to 7 days' storage, based on manufacturer claims, compared to the lower stability of the cfDNA collection tube from Roche Diagnostics GmBH. Regarding the centrifugation conditions, such as a double-spin plasma preparation protocol followed by a second high-speed centrifugation step at 16,000 g (3,000–16,000 g) for 10 min, this influences the presence of high-quality cfDNA purification [28, 88, 102]. The third critical factor leading to preanalytical biospecimen handling is the type of cfDNA purification kit. Tables 2 and 3 list the broadly utilized extraction kits and techniques. A parallel review between several different kinds of cfDNA extraction kits and procedures reported the highest purified cfDNA outputs with the Norgen Kits. However, depending on how well the beads or silica gel membranes work at capturing DNA during the purification process, these different extraction tools may make different parts of plasma cfDNA molecules that are of different sizes. It is also important to think about the possibility that differentially high-molecular-weight DNA from necrotic malignant tumor cells could be kept in the extracted combined cfDNA eluate using a number of different kits that are compatible with other extraction methods. The molecular mechanisms underlying nucleosome release into circulation are associated with apoptosis-dependent cell death upon targeted therapies like tyrosine kinase inhibitors. This information must be taken into consideration when tracking mutations on cfDNA for therapeutic drug monitoring [104].

More recently, tests comparing six different magnetic-bead-based cfDNA extraction platforms showed that the high-throughput cell-free circulating DNA isolation kits from 4 ml of plasma by QIAGEN and Norgen Biotek's companies were better at recovering DNA fragments with a length range of 50–808 bp. While the Applied Biosystems isolation kit (wide sample volume inputs range from 500 *µ*l to 10 ml of plasma or serum) leads to no carryover of shorter size fragments than 50 bp relative to other reliable separation strategies associated with PerkinElmer kits that delivered significantly efficient retrieval of DNA molecules in the size range of 75–300 bp from 0.5 to 1.5 ml of plasma or serum samples [28, 105, 106]. For systemic cfDNA methylation profiles, a potential adapted MethylMiner (Invitrogen) method for the extraction of plasma-derived methylated cfDNA variants has been described. Another considerable preanalytical variable is deterioration due to long-term cfDNA storage at −80°C. The incorporation of bisulfite conversion treatment is also an important preanalytical factor. Currently, many kits are employed for methylated DNA molecular pattern studies, but one product that is most widely fitted for the bisulfite conversion process of plasma-based cfDNA is the InnuCONVERT Bisulfite Body Fluids Kit (Analytik Jena AG), which performs with a maximum 3 ml plasma input volume [28]. The Epitect (Qiagen) Kit is best suited for efficient bisulfite conversion performance on purified cfDNA with “a limited quantity” of fragmented DNA. MOB is a new integrated sample extraction and processing technique that facilitates cfDNA isolation and bisulfite treatment for up to 2 ml of plasma or serum and guarantees great recovery and assay sensitivity of the CpG methylation status analysis.

Systemic chronic stress from physical or mental events and acute stress conditions like intense exercise followed by muscle injury and repair responses cause DNA to be released over a long period of time. Following this, there is a significant increase in cfDNA levels during cell death or apoptosis and changes in cfDNA methylation profiling In addition, acute plasma viral reactivation ratios for HIV, hepatitis B, and Epstein–Barr infections lead to high levels of cfDNA because there is a lot of viral DNA that is not in cells. Clinical factors like clinical stage, histopathological grading, primary, local, or distant recurrence pattern, and response rate to targeted therapy are likely to have additional effects on the amount of tumor-of-origin cfDNA, which reveals more uses for cfDNA tests in clinical diagnostics and prognosis. Similarly, a major potential association between circadian rhythmicity and a more elevated cfDNA amount at midday was recognized in patients with stages I–III CRC compared with cases with stage IV [35]. The genotoxic effects of environmental agents like pesticides on the increase in cfDNA concentration were described previously, as exposed women harbored a higher cfDNA level than exposed men [107]. Age can also be an especially significant predictor when one surveys relative input amounts of cfDNA at basic genomic sites, in particular, transcription initiation and termination regions, or organ-specific and cfDNA-correlated methylation footprints [27, 28]. Conclusively, different studies demonstrated that both genomic and environmental factors modulate an individual's cfDNA level, which is therefore highly variable in the healthy population. Our study suggests that the diagnostic sensitivity of cfDNA evaluation as a noninvasive biomarker could be improved if the person's cfDNA level is known prior to disease onset or cancer presentation. If further verified in larger cohorts, plasma cfDNA levels could thus serve as a sensitive, noninvasive personalized biomarker for the diagnosis and prognosis of many diseases, particularly cancers. On the other hand, not alone but in combination with CTCs, they can improve the diagnostic and prognostic applications of cancer. For example, concluded that the combined analysis of cfDNA and CTCs provides some extra information to detect patients with a worse prognosis, as the sensitivity to detect relapses increased from 79% to 90% within 2 years. Another study using a PCR-based NGS assay proves this combined analysis method is useful in cancer diagnosis. The high integrity of miRNAs in circulation blood contributes to establishing miRNA expression patterns and takes advantage of its potential for reliable spotting over combined assay-based TRIzol material isolation procedures with spin columns, marketing accessible kits (Table 4), and immunomagnetic beads coated by capture antibodies for exosomal miRNAs. Although the coextraction TRIzol protocol for selective circulating miRNA isolation has the possibility of cross-reactivity with DNA, lipids, or proteins and cell-derived microparticles like platelets or erythrocytes, besides phenolic contaminants [12, 112].

## 7. Cancer Targeted Genotyping: Progression of Individualized CRC Therapies by Concentrating on Actionable Genes and Regions

### 7.1. Investigation of Genetic Variations, MSI, and CNVs of CRC

#### 7.1.1. Investigation of Genetic Variations, MSI, and CNVs of CRC in Blood

**Figure 1:**
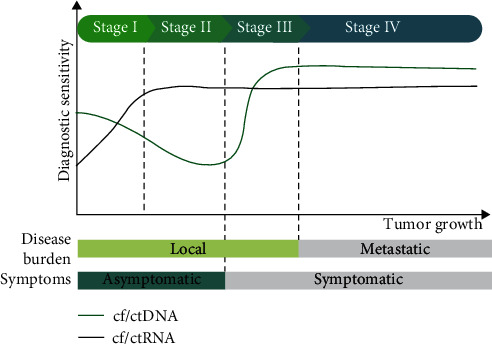
Diagnosis of CRC utilizing circulating tumor nucleic acids. According to a robust, sensitive, and specific noninvasive screening associated with a panel of elevated ctDNA and ctRNA signature expression, it is conceivable that these blood biomarkers can be used for early CRC detection.

Microsatellites are tandem repeats located throughout the genome. Microsatellite instability (MSI is the deletion or insertion of microsatellite repeats that is associated with a genetic instability in 15% of all CRCs and resistance to chemotherapeutic agents (more sporadic than hereditary nonpolyposis) and is due to faulty DNA mismatch repair (MMR) genes. Various researchers are interested in the influence of cfDNA in conquering the tumor mutational burden from two CRC MMR-D subjects, which clarified the feasibility of cfDNA as a substitute marker for MMR-D and the five quasimonomorphic MSI markers BAT-25, BAT-26, NR21, NR24, as well as NR27, and four MMR genes (MSH2, MSH6, PMS2, and MLH1). MSI-H CRC cases guarded from the immune checkpoint barricade displayed comobilization of CTNNB1, APC, and/or RNF43 mutations referring to the WNT/beta Catenin signaling. There has been a serious demand for detecting predictive molecular markers for chemotherapy response (sensitivity or resistance) both in adjuvant and metastatic settings. Signature molecular markers that have been seriously investigated involve thymidylate synthase (TS) expression, and upregulated ERCC1, among others. Most recently, MSI has also been displayed to predict failure of response to adjuvant 5-FU in stages II and III CRC cases (and feasible side effectsin stage II cases). However, in the MRC FOCUS trial of metastatic CRC, mutant BRAF was not presented as an accurate predictive biomarker for any 5-FU-based chemotherapy regimen [116–119]. Studies from China characterized CRCs according to their MSI statusctDNA, and their amplicon-based NGS data provided essential intelligence about the power of MSI from ctDNA as noninvasive prognostic and diagnostic markers among CRC subjects [120]. One study on point mutations of cfDNA demonstrated serum-sensitive detection rates of genes TP53, APC, and KRAS were 34.2%, 30.4%, and 34.0%, respectively, for recognizing rest illness after surgical resection. These personalized mutations can avert the progression of the array housing sum of the somatic mutations inexpensively (Table 5 and Figure 1).

## 8. Examining Genetic Variants, MSI, and CNVs in CRC Plasma

The occurrence of KRAS, BRAF, IDH1, IDH2, PDGFRA, and TP53 mutations following whole genome sequencing testing of cfDNA was found to be a more popular etiologic biomarker for genomic instability in CRC, influencing about 75%–85% of cancers. Recently, ERBB2 amplification has also been considered as a detection process for CRC, with a sensitivity of 91.7% and a specificity of 88.9% (Table 4 and Figure 1) [149–153].

### 8.1. Circulating mRNA Indicators

#### 8.1.1. Circulating mRNA Indicators in Blood

In CRC, circulating mRNA biomarkers such as epidermal growth factor receptor (EGFR), cytokeratin 20, and CEA can be identified differently from other blood cells by multiplex RT-qPCR-based telomerase reverse transcriptase (TERT) [154–156]. Among other diagnostic and prognostic panels, LMNB1, VNN1, IL2RB, CLEC4D, ANXA3, TNFAIP6, and PRRG4 circulating mRNA biomarkers can be introduced from numerous past studies. Actually, the accuracy of all these proposed screening procedures has already been verified across different populations. For example, 202 CRC Canadian patients showed a diagnostic sensitivity of 72% and a specificity of 70% compared to 208 healthy individuals, while in 99 Malaysian CRC patients, a sensitivity and specificity of 61% and 77%, respectively, were determined compared to 111 controls (Figure 1).

### 8.2. CRC Epigenetic Signatures

#### 8.2.1. Promoter Methylation in cfDNA/ctDNA in Blood

DNA methyl transferases (DNMTs) catalyze DNA methylation, which is the covalent transfer of a methyl group to the C-5 position of the cytosine ring to produce 5-methylcytosine. Methylation at CpG dinucleotides of regulatory regions leads to the suppression of gene transcription, an early event detectable in tumor suppressor genes of cfDNA in cancer patients [157, 158]. Mutation detection has to look for changes all over the genome. Methylation analysis, on the other hand, may be easier because it only needs to look at changes in the promoter region [114]. Compared to other mutational events, CpG island hypermethylation is more common to detect earlier stages of CRC and precancerous polyps. However, it might be present in some, but not all, malignancies, limiting the accuracy of discrimination between different types of cancers [85]. In various cancers, extensive CpG methylation patterns in cfDNA increase the chance of ctDNA detection and diagnosis using blood samples. According to tissue-specific methylation patterns, the origin of the tumor can also be identified with high sensitivity and specificity [36]. Due to the tumor-specific characteristics of ctDNA, including mutations and epigenetic variations, the early hypermethylation pattern of tumor suppressor genes in the promoter region can be used as a cancer detection biomarker. There are two methods to characterize DNA methylation in cfDNA: qPCR-based methods to detect specific regions and deep sequencing-based methods to reveal DNA methylation profiles in the whole genome. Bisulfite sequencing, which is highly utilized in cfDNA methylation profiling, is not cost-effective. However, methylated DNA immunoprecipitation followed by high-throughput sequencing (methylated DNA immunoprecipitation (MeDIP)-seq) is a genome-wide and cost-effective method that is hardly used for cfDNA characterization. MeDIP-seq has been performed in a 2019 study to evaluate the biomarkers of lung cancer. Methylation of B3GAT2 has been used as a biomarker in CRC diagnosis [2]. There is a correlation between the colorectal CpG island methylator phenotype (CIMP) and DNA methylation of the MLH1 (MutL homolog 1) promoter, BRAF mutations, microsatellite instability, and somatic mutations in KRAS (62). In tumor suppressor genes such as O6-methyguanine-DNA methyl transferase (MGMT), bone morphogenetic protein 3 (BMP3), and EFHD1 (EF-hand domain family member D1), methylation at certain CpG sites results in gene suppression and the formation of malignancy. Therefore, using noninvasive methods such as methylation-specific (MSP) polymerase chain reaction (PCR) on cfDNA can lead to a better prognosis and disease management as a novel serum biomarker [3]. A new study was designed to examine the utilization of ctDNA methylation markers for the diagnosis and prognosis of CRC using a prospective cohort to evaluate their efficiency in screening patients susceptible to CRC development. By comparing CRC tumors to normal leukocytes, a CRC-specific methylation pattern was identified, and an algorithm was applied for a predictive diagnostic and prognostic model for cfDNA obtained from a cohort of 801 patients with CRC and 1,021 normal controls. The power of this model for discriminating CRC patients from normal controls was very promising (area under curve = 0.96). The prognosis and survival of patients with CRC (*p*  < 0.001) were also accurately predicted by the prognosis prediction model. Moreover, other cfDNA biomarkers with abnormal DNA methylation, including genes of TPEF/HPP1, ALX4, TMEFF2, NGFR, NEUROG1, FRP2, APC, MLH1, RUNX3, and CDKN2A/P16h, have been indicated to be extremely sensitive and specific in CRC patients, along with methylated HLTF, as powerful prognostic biomarkers associated with tumor stage and size, metastatic disease, and recurrence of illness. Supplementary, methylated DFNA5, and HPP1 also have prognostic qualities [159–163]. A classification of CRCs was performed based on ctDNA markers using a clustering method, and two classes of CRC patients were obtained with significantly different survival rates (*p*=0.011). A single ctDNA methylation marker named cg10673833 was also introduced with appealing sensitivity (89.7%) and specificity (86.8%) to diagnose CRC and precancerous lesions in a prospective cohort [130, 164].

#### 8.2.2. Promoter Methylation in cfDNA/ctDNA in Plasma

The combination of different methylation targets in cfDNA can lead to effective biomarkers for the early diagnosis of CRC. The most studied plasma-based epigenetic marker for CRC screening in cfDNA is methylated Septin 9 (mSEPT9), which was found to be cost-effective compared to the absence of screening [165]. About 8,000 asymptomatic patients from the US and Germany were studied with routine screening colonoscopy in combination with SEPT9 analysis in the blood. The sensitivity and specificity of SEPT9 DNA methylation in cfDNA were 48.2% and 91.5%, respectively [165]. In a recent study on tumor liquid biopsies, investigating mSEPT9 in CRC patients resulted in a 72% diagnosis of stages I–III cancers with 93% specificity [8, 166]. A few studies have evaluated the epigenetics of glycan genes, such as the methylation status of beta-1,4-galactosyltransferase1 (B4GALT1) in cancer cells. This type II membrane-bound glycoprotein interacts with EGFR and inhibits the dimerization of the receptor and its signaling pathway in human hepatocellular carcinoma cells. In a 2019 study, the diagnostic, prognostic, and therapy-response predictive power of the glycogen B4GALT1 was investigated in CRC patients. Quantitative methylation-specific PCR (QMSP) was used to detect hyper methylated B4GALT1 and dd-QMSP in plasma in four cohorts of metastatic CRC cases. Promoter hypermethylationand downregulation of B4GALT1 expression indicated poor prognosis, decreased cetuximab response, and liver and lung metastases in CRC. B4GALT1 can be a sensitive biomarker for the diagnosis of CRC and the prediction of drug responses [85].

### 8.3. miRNAs Markers of CRC in Plasma

MicroRNAs (miRNA) are brief (18–25 bp in length), ribonuclease-protected, noncoding copies that function as gene modulators (tumor suppressive versus oncogenic) with potential diagnostic, prognostic, and therapeutic functions [167]. Comparative analyses between different species demonstrate that miRNAs are evolutionarily conserved and play important roles in a wide variety of cellular physiological and pathological processes [168]. Currently, it is established that any miRNA acting as a primary regulator is capable of regulating the gene expression of significant quantities of targeted mRNA [169]. Multiple miRNA genes are located in chromosomal regions that undergo translocations, deletions, or duplications, resulting in the production of atypical expression templates in multiple tumor types, particularly CRC. miRNAs are reassuring as noninvasive biosignatures in the circulation, unlike mRNA, due to their resistance to extra-exosome ribonuclease and resistance to high pH levels. With a sensitivity and specificity of 83.3% and 69.0%, respectively, miR-760 and miR-601 were found to have lower expressionin advanced adenoma (AA) and CRC cases compared to normal specimens after contouring 742 miRNAs on CRC trials and normal specimens. miR-532-5p, miR-331, miR-335, miR-19, miR-142-3p, miR-29a, miR-19b, miR-15b, miR-18a, miR-17, miR-652, miR-532-3p, and miR-19a were identified as candidate miRNAs in plasma or serum by two additional research groups, with a sensitivity of 78.6% and a specificity of 79.3%. The same set designed a three-serum miRNA pattern, miR-139-3p, miR-431, and miR-15b, with a sensitivity of 93% and a specificity of 74% for discriminating between patients with stage IV CRC and normal specimens. Ahmed et al. [170] stated to verify a 15-panel miRNA of that nine (miR-214, miR-183, miR-92a, miR-196a, miR-20a, miR-17-3p, miR-7, and miR-21) were overexpressed and six (miR-138, miR-146a, miR-222, miR-127-3p, miR-143, and miR-124) were suppressed in CRC cases' plasma and tissue with 90% sensitivity and 95% specificity [170–172]. Numerous aggregated diagnosis studies focus on miRNA-based early screening for AA and CRC (sensitivity and specificity ranging from 78% to 93%, 41% to 95%). Harlé [8] validated a set of 15 miRNAs, of which nine (miR-7, miR-17-3p, miR-20a, miR-21, miR-92a, miR-196a, and miR-214) were upregulated and six (miR-124, miR-127-3p, miR-138, and miR-222) were downregulated in the plasma and tissue of CRC patients. Multiple research groups have extracted their candidate miRNAs from the scientific literature. One study confirms miR-29a and miR-92a on 120 CRC, 37 AA, and 59 healthy individuals to differentiate between CRC and healthy individuals with a sensitivity of 83% and a specificity of 84.7% [173, 174]. miR-221 was found to be upregulated in CRC with a sensitivity of 86% and a specificity of 41%, as validated on a cohort of 103 CRC and 37 healthy controls [175]. Detection of CRC at an early stage is predominantly investigated in patients of African–American descent; sensitivity and specificity range from 78% to 93% and 41% to 95%, respectively. Several MicroRNA biomarkers, including miR-15b, miR-17-3p, and miR-18a have been proposed. However, other scientists have not validated every single one of these biomarkers on account of variations in the patient population, endogenous controls, or instrumentation. Additional assessment and verification of these sets of microRNAs is required [176, 177].

### 8.4. Long Noncoding RNAs Markers of CRC in Plasma

EDTA plasma-represented cell-free lncRNA biomarkers can have a diagnostic potential for targeted clinical management of diverse forms of cancer. One of the leading results is drawn from the upregulation of circulating lncRNA HULC in subjects with hepatocellular carcinoma. The plasma review of a series of diagnostic lncRNA biomarkers, including PTEN1 (phosphatase and tensin homolog1), long stress-induced noncoding transcripts 5 (LSINCT5), urothelial carcinoma-associated 1 (UCA1), cancer-upregulated drug resistance (CUDR), and H19, showed a great upregulation in gastric tumor cases and approved lncRNA POU3F3 expression in serum coupled with the plasma status of squamous cells can strengthen screening productivity for timely identification. To date, a little data have been released about the expression of circulating lncRNAs as a potential noninvasive biosignature for early CRC detection. For example, increased CRNDE-h transcripts have a sensitivity of 87% and a specificity of 93%, besides their high expression of plasma-based HOTAIR and CCAT1 in CRC cases compared to normal cases. This combined association revealed the largest diagnostic quality, with 84.3% sensitivity and 80.2% specificity, for effective detection of CRC at an early stage [178–182].

## 9. Application of CNAs to Determine Minimal Residual Disease and Evaluate the Effectiveness of Adjuvant Therapy in CRC

**Figure 2:**
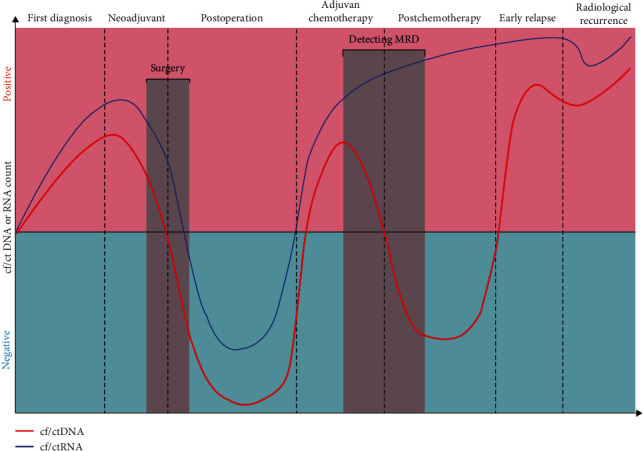
CRC prognosis and treatment response based on circulating tumor nucleic acids throughout the duration of the initial CRC diagnosis, ctDNA, and ctRNA levels were elevated. Then, ctDNA and ctRNA rapidly disappear from the peripheral blood after surgery, but they rise again, resulting in an early relapse. In the absence of tumor biopsy specimens, they can also be used for cancer molecular tracking to detect MRD, monitoring therapeutic response, and predicting the risk of cancer recurrence. It was also correlated with the worst prognosis in advanced stages of colorectal cancer. In addition, there is evidence that ctDNA predicted future radiographic relapse owing to a small decrease and increase in the blood extents, respectively, after adjuvant therapy.

The detection of residual CNAs after a surgical intervention or curative-intent therapy may indicate the presence of minimal residual disease (MRD), which could distinguish patients with a high risk of relapse (Figure 2). The CtDNA methods used in the major prospective surveys in cases of resected CRC belong to two main groups: (a) cancer-agnostic techniques and (b) cancer-informed techniques are extensive panel-based sequencing methods utilized without background information about the case's cancer mutational profile and intended to search for genomic changes and unique DNA methylation patterns recognized to appear in a given cancer type (e.g., Guardant REVEAL) [183]. Cancer-agnostic methods have various advantages that involve the ability to use the examination, logistical simplicity, fast turnaround time if the primary cancer tissue is not accessible, and the possibility of evaluating MRD regardless of the clonal progression of the micrometastatic cancer cells. Conversely, tumor-informed assays need background information on the cancer genomic profile of the index patient, commonly obtained by whole-exome sequencing or directed sequencing of the primary cancer (e.g., SignateraTM, SafeSeqS) [24]. These assays are personalized and intended for each case to detect case-specific genomic changes through the directed sequencing of the plasma DNA and ddPCR platform [184]. Cancer-informed techniques also have various advantages and disadvantages, respectively, involving a maximum rate of methodical sensitivity low to a VAF of 0.01% and a decreased rate of false-positive outputs secondary to clonal hematopoiesis of indeterminate potential (CHIP), require a longer turnaround time, incur additional costs for tumor sequencing, weakly capture all MRD-specific changes relevant to intratumoral heterogeneity, and probably not diagnose primary mutations resulting from treatment-associated selection pressure [184–186]. Tracking of ctDNA determined a promising medicine's potential to identify MRD for solid tumors after initial treatment with surgery and in the progression of radiological tumor relapse [187]. The presence of ctDNA involving somatic genomic alterations identified in an individual's cancer is a specific signal of survived hidden cancer cells following surgery. It is exactly when surgical removal of the primary tumor together with postoperative ctDNA results in an early residual metastatic disease, which is correlated with the increased likelihood of recurrence [188, 189]. MRD evaluation through centralized ctDNA testing is associated with a poorer prognosis in subjects with various types of solid tumors. Because of the low plasma ctDNA amount correlated with MRD, quantitative techniques should potentially serve for the diagnosis of genomic variants at a VAF ≤ 0.1% [190]. In subjects with stage II CRC (approximately 25% of all CRC), Chen et al. [191], utilizing a broad panel-based NGS to quantify ctDNA, released one large-scale prospective cohort study relating to patients with TNM stage II/III (*n* = 276), illustrating that ctDNA expressively outperformed signature clinic pathologic parameters as a prognostic indicator. Peripheral blood mononuclear cells, plasma, and surgically resected tumor specimens were assessed for consecutive ctDNA monitoring in each subject. Among 112 subjects with surgically TNM stages II CRC, the presence of ctDNA in postsurgery plasma specimens was significantly associated with recurrence-free survival (RFS) correspondence to the adjuvant chemotherapy: ACT-benefit and ACT-futile subgroups, signifying that ctDNA content remained positively the strongest independent predictor of RFS. All 174 ctDNA-positive patients (II + III) who benefit from ACT had an expressive susceptibility to cancer recurrence (hazard ratio—HR 9.99; 95% confidence interval (CI) 4.40–22.69; *p*  < 0.001). Similarly, the 2-year RFS rate of 89.6% for patients with undetectable postoperative ctDNA who received adjuvant chemotherapy (95% CI 84.5%–95.0%) was close to that of undetectable postoperative ctDNA patients who did not receive any adjuvant chemotherapy (89.2% (95% CI 81.4%–97.8%)). Actually, they observed a significant high radiologic recurrence likelihood in ctDNA-positive CRC patients with clinical stage II who were not treated with ACT postoperatively. This likelihood is higher than in cases with clinical stage III CRC, who are usually cured with adjuvant chemotherapy. Conversely, patients with negative ctDNA had a low risk of radiologic recurrence postoperatively (HR 12.76; 95% CI 5.39–30.19; *p*  < 0.001), with a duration of 2-year RFS frequency of 87.7% (95% CI 81.5%–94.2%), implying a subgroup where ACT was unlikely to be beneficial. Postoperative ctDNA-positive patients predicted a very poor RFS duration of 2 years (25.0% (95% CI 9.4%–66.6%)). These results proved that ctDNA could be applied to screen for MRD in primary colorectal tumors. Conclusively, in people with stage II CRC, ctDNA probably may be a productive prognostic indicator postoperatively and might direct primary adjuvant therapy. Postadjuvant chemotherapy risk stratification and monitoring are also required, but making better decisions is currently lacking. Chen et al. [191] further manifested in patients with clinical stage III CRC that detectable ctDNA at postoperative and after completion of adjuvant chemotherapy prognosticated a very high cumulative radiological recurrence due to clinical recurrence. When compared to radiological recurrence, ctDNA profiling had a median lead time of 3–7 days, which could improve the stratification of postoperative risk and facilitate clinical decision-making for pathologic stages II and III CRC patients. Individualized serial analysis of ctDNA during or following adjuvant chemotherapy regimens may also be an early real-time indicator of ACT outcome. Interestingly, the consecutive ctDNA positive subjects had significantly increased metastatic recurrence likelihood with a 2-year RFS frequency of 24.0% (95% CI 11.9%–48.2%) despite receiving ACT, although just four out of 100 subjects with consecutive positive ctDNA findings experienced a 2-year RFS frequency of 96.0% (95% CI 92.2%–99.9%) (HR 32.02; 95% CI 10.79–95.08; *p*  < 0.001). Recent data also suggest that integration of epigenomic markers, such as DNA methylation analysis in plasma samples, probably promotes MRD evaluation sensitivity over routine genomic change evaluation assays alone, and the incorporation of genomic and epigenomic detection enhances application. Interestingly, routine serum CEA levels did not predict recurrence (hazard ratio 1.84 (*p*=0.18); PPV = 53.9%) [190]. Approximately 30% of CRC originates from rectal sections, and preoperative chemo-radiotherapy (CRT) is the routine therapy for locally advanced rectal cancer (LARC), while the treatment response to CRT differs from perfect to weak [192]. However, the nonreceiver group is susceptible to ineffective, hazardous treatment, while the group with a pathological complete remission following CRT exhibits better patient outcomes in comparison to nonresponders. In LARC patients, serial ctDNA profiling was also a significant predictor of early recurrence immediately after surgery [193]. Accordingly, the study of Rampazzo et al. [194] that was carried out on cases with primary rectal adenocarcinoma who were at diagnosis pre-CRT (T0), 2 weeks following starting CRT (T1), after CRT and before (2–0 days) surgery (T2), and 4–8 months subsequent to surgery (T3) revealed that the amounts of circulating cell-free TERT mRNAs and their genetic variations before or following neoadjuvant chemotherapy can be considered as a complex of individual predictive factors that are associated with the patient's response to the CRT therapy. The area under the curve for the prediction model was 0.80, with a 95% CI of 0.73–0.87. They also proved that patients with measurable circulating TERT amounts at the continuous time of T2 and T3 showed a higher risk of progression to the severe stage of the disease by 2.13-fold (95% CI 1.10–4.11) and 4.55-fold (95% CI 1.48–13.95), respectively, than those with unmeasurable plasma TERT amounts. TERT levels at the continuous time of T2 and T3 were menacingly correlated with PFS in the univariate statistical analysis. The 5-year PFS of the cases with observable T2 TERT amounts were 58.8% (95% CI 47.1–68.7) and 79.9% (95% CI 67.8–87.8) for a median follow-up of 61.2 months. The 5-year PFS of the cases with observed or disappeared T3 TERT amounts in plasma were 53.1% (95% CI 37.6–66.3) and 90.6% (95% CI 76.6–96.4), respectively (*p*  < 0.0001).

## 10. Conclusion and Future Outlook

**Figure 3:**
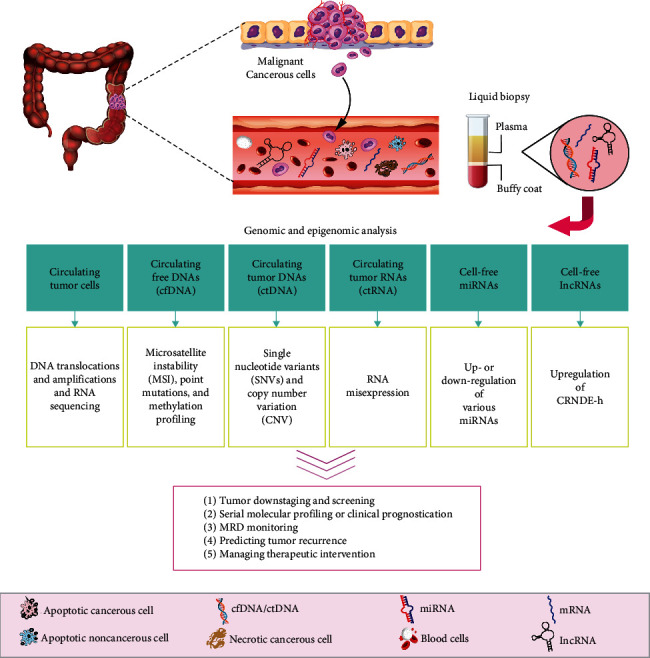
The clinical significance of tumor-derived circulating nucleic acids in advanced colorectal cancer. This diagram depicts the most important clinical applications of liquid biopsies. These include tumor genotyping and epigenotyping in the diagnosis of CRC, evaluating drug response, tracing minimal residual disease, and observing clonal evolution.

Liquid biopsies provide an excellent opportunity for early cancer detection and posttreatment patient monitoring. The use of free CNAs in the blood is extremely feasible and reproducible, by contemplating its relevance in genetic and epigenetic alteration detection. Several genetic and epigenetic markers have been proposed and can be used individually or collectively in the management of patient cases. Recent advances have enabled the extraction of CNAs from the bloodstream. We have outlined the pros and cons of using plasma versus serum, one technique versus another, and one bioinformatics tool versus another. Further advancements in isolating circulating tumor cells from blood samples will significantly reduce the amount of blood-contaminating nucleic acids and increase the specificity of cancer cell-specific nucleic acids, thereby enhancing the potential use of liquid biopsies. Consequently, CtNAs are noninvasive for early CRC diagnosis, prognosis, MRD detection, and treatment response (Figure 3).

## Figures and Tables

**Table 1:** The challenges of current semiautomated strategies for CTCs extraction and molecular quantification.

Methods	Challenges	References
The cell search system	Low sensitivity (false negative) due to only EpCAM-positive CTCs consideration or missing CTCs subclusters engaged in EMT, low specificity (false positive), small productivity, and diverse specimen testing for direct recognition and evaluation of intact cells in the epithelial cellular adhesion molecule (EpCAM)-based cell search R system	[24]

Microfluidic-based positive enrichment technologies	Complete separation proficiency by a single or multiple capture antibodies for pathology-specific target cells in small fluid volumes after laminar flow without pretagging	[25–27]

DNA or RNA sequencing	Absence of smaller CTCs or CTC fragments as a result of a false-positive test	[28–30]

Biophysical CTCs extraction	Requirement of spiked-in experiments for validation of different CTC isolation approaches prior to more accurate clinical reliability for removing interference with large leukocytes (e.g., monocytes) and CTCs due to their small size and low frequency of size-based techniques below 10%	[28–30]

**Table 2:** Comparing epigenetic methods for CRC diagnosis and treatment.

Methods	Advantages	Limitations	Bioinformatics tools
Gene methylation analysis	Detects earlier stages of CRC and precancerous polyps with high sensitivity and specificity [35], genome-wide DNA methylation analysis with using NGS or microarrays [36]	Limited accuracy of discrimination [36]	ENCODE, modENCOD, Econsortia, QDNAseq, CancerLocator, CancerDetector [37]

Multibiomarker fecal cfDNA assays			
Modified solid-phase minisequencing method, QuARTS method, and hemo quant test, oligonucleotide-based hybrid captures-based real-time PCR methods	A promising approach toward cost-efficient DNA diagnostics and comparative sequence analysis with high sensitivity (85%–91%) and specificity (60%–93%) comparable to that of FOBT for early detection and screening CRC [38–40]	Not declared	UMI-tools, MAGERI [41]
Infinium HumanMethylationEPIC BeadChip microarray	Increased genome coverage of regulatory regions, high reproducibility and reliability, easy to use, time-efficient and cost-effective [42]		
Whole genome bisulfite sequencing (WGBS)	Single-nucleotide resolution of DNA methylation across the entire genome [43]	Not declared expensive, time consuming, and nonapplicable for high throughput [44]	Wg-blimp, msPIPE, MethGo [45–47]

qRT-PCR	Facility, quantification, high reproducibility, and accuracy similar to NGS and the agilent microarray [48–50]	Limited potency for low-expressed miRNAs detection, contamination, less precise in regions of homopolar (similar) bases [48–50]	QDNA-seq, WisecondorX, BIC-seq2, CNVkit [51–53]

RNA sequencing	Direct adapter-dependent ligation of less short noncoding RNAs (e.g., miRNAs, piRNAs, and endosiRNAs,) using NGS [54], cheaper, quicker, more accuracy compared to qPCR or Sanger sequencing [55]	Bias resulting from the impact of sequence on ligation especially for 5′ adapter ligation vs 3′ adapter ligation and contaminant PCR products devoid of each inserts [54]	FastQC, Cutadapt, STAR, HTseq [56]

**Table 3:** A cross-platform comparison of sensitive ccfDNA detection techniques for CRC analysis.

Methods	Application	Advantages	Limitations
SeqCap EZ HGSC VCRome®	Differential presence of exons (DPE) detection and tumor mutation load of plasma cfDNA by WES [57, 58]	High-throughput screening alternative of target regions up to 7 Mb, cost effective, limiting the risk for incidental findings, and increasing sensitivity and specificity rates [59]	Require certain equipment such as the hybridization station [59]

QX200 Droplet Digital PCR System®	Quantified detection of low-frequency alleles within a limited cfDNA pool [58]	Absolute count of target nucleic acid copies per sample volume, most commonly copies per microliter. Superior accuracy and partitioning [60]	Droplet variability in size and shape adversely affect robustness and reproducibility [61]

Guardant360® assay	Tumor profiling by liquid biopsy for monitoring and aftercare of a cancer therapy [62]	Comprehensive genomic profiling in patients with advanced solid tumors [63]	Detecting somatic cfDNA mutations that simultaneously exist in the blood lineage [64]

ProFlex PCR system®	The method of KRAS mutations analysis using loading dPCR reaction admixture over a QuantStudio 3D Digital PCR 20K Chip v2 [65]	Partitioning of a reaction into nanoliter reaction chambers by a microfluidic device, more sensitive for strain's specific detection with less variability than qPCR [66]	Not declared

Commercial KRAS Screening Multiplex®	The sensitive and quantitative QX100 droplet digital PCR for multiplex ready-to-use KRAS somatic mutation detection [67]	Good sensitivity and specificity and good concordance with conventional clinical mutation testing of archival specimens [68]	High cost and high DNA input requirements [69]

Onco BEAM-TM-RAS-CRC™®	Highly sensitive and quantitative digital PCR platform for screening somatic KRAS and NRAS mutations by flow cytometry [67]	Reliable detection of mutations from cell-free DNA that occur at mutant allele frequencies (MAF) as low as 0.01% [70]	Not declared

56GOncology Panel®	The multiple targeted next-generation sequencing library preparation [73]	Comprehensive and hotspot coverage of 56 clinically relevant, oncology related genes [71]	Not declared

Quant Studio 3D Digital PCR 20K chip®	The detection of the mutational spectrum of cfDNA [72]	Less pipetting steps and to reduce PCR contamination accuracy and precision to quantification of cfDNA [73]	Performing only one sample per chip, and two probes per chip in multiplex fluorescence [73]

Ion AmpliSeq Library and IonAmpliSeq ®LibraryIon 318 Chip®	To detect cancer-specific mutations of ccfDNA using the Ion Chef System [72]	Targeted gene sequencing with 4–5.5 million reads per run, easy handling and loading of templated products for sequencing, compatibility with current library preparation methods [74]	Not declared

Illumina HiSeq 2500 ®	The ultra-deep targeted sequencing [75]	Low instrument costs and a small instrument footprint, all while maintaining the high data accuracy of SBS [76]	No long lengths of DNA sequences can be obtained using these methods [77]

BD Accuri C6®	The interrogation of harvested beads by fluorescent probes that specifically hybridize to either methylated or nonmethylated derived sequences within the queried sequence [78]	Reliable instrument performance with automated QC, volumetric counting and continuous sampling [79]	Not declared

Invader Plus assay®	Invader Plus assay with peptide nucleic acid clamping for KRAS mutations status [80]	Significantly more sensitive as more than 10^7^ reporter molecules were generated per target molecule in a 4 hr reaction. No longer requires the synthesis of allele-specific labeled oligonucleotides [81]	Less-abundant targets mostly require PCR-driven preamplification [82]

Biocartis Idylla™, Roche COBAS z480® Sysmex Inostics BEAMing®	Three commercially available PCR-based platforms for detection of hotspot mutations in KRAS [83]	Fast and accurate detection of KRAS mutations by a sensitive and specific standardized cost-effective method, easy to implement in settings with limited expertise in molecular diagnostics [87]	They are single-gene tests and therefore only a few genes can be analyzed [84]

QX1000 Droplet Generator DG8 Cartridge System®	To analyze the hyper methylated genes in plasma cfDNA based on the droplet digital quantitative methylation-specific PCR (dd-QMSP) [85]	Partitions each sample into 20,000 uniform nanoliter-sized droplets in which nucleic acid molecules are distributed in a random fashion [85]	Not declared

ColoDefense assay®	New blood-based methylation assay for disease screening [86]	Excellent sensitivity and specificity for combined detection of multiple biomarkers in the same run and avoidance of repeated blood draws [87]	Not declared

**Table 4:** Methods for obtaining cfDNA from plasma and serum.

Methods	Approaches	References
QIAamp circulating nucleic acid kit	Easily extraction of ccfDNA with rapid spin-column or 96-well-plate technique without dependent phenol-chloroform extraction	[75, 108]

Triton/heat/phenol protocol (THP)	Greatest efficiency, cheaper, and high-quality outputs for further plasma cfDNA isolation vs. the Qiagen kit assay	[109]

NucleoSpin® Plasma X'S	Very fast extraction of this method leading to a much integrity DNA produce that may utilize for the recovered of small DNA fragments	[110]

Maxwell® 16 LEV DNA purification kit	Automated purification of cfDNA from much less 10^4^ cells using silica-coated paramagnetic particles (PMPs), as a mobile solid phase for optimizing of the capture, washing and elution of the target material	[111]

**Table 5:** Comparison of genetic methods for CRC diagnosis and screening.

Methods	Advantages	Limitations	Bioinformatics tools
ALU-based *q*-PCR	Acceptable specificity, diagnoses CRC patients from healthy individuals [121] (screening and prognostic value for CRC disease)	Low sensitivity for differential diagnosis of stage I/stage II CRC and adenomas [121]	REST, pyQPCR, PrimerPy, GenEx, MultiD PowerNest, qBASE+ [122]

KRAS mutation analysis	Exists in 40% CRC cases mostly in codons 12 [36], negative predictive factor for metastatic CRC to treat with anti-EGFR antibodies [123], and correlates with the prognosis of the disease [124]	Not declared	COSMIC, TCGA, GSEA, GO enrichment, KEGG, STRING, CPTAC, [125–127]

NGS assay: (1) Safe-SeqS (2) TEC-Seq	Detects somatic mutations in CRC early stages [8], prognosis and prediction to anti-EGFR antibodies or drug selection [8]	Not declared	Ion Torrent, Illumina, Read Filtering and Trimming, Burrows–Wheeler transform algorithm, Torrent Mapping Alignment Program, SAMtools Genome Analysis Toolkit (GATK), and Picard [128]

BRAF mutation analysis	Differentiating CRC from precancerous or state and distinctive histologic subtype of CRC [129], correlates with poor prognosis of the disease [130], and predictive biomarker for targeted therapy and prognosis [95]	It exists in 5%–10% of CRC tumors and should be used with other markers [131]	CBioportal, oncomine [132]

APC mutation analysis	Exists in 85% of colorectal tumors and 60% of stage I/II CRC patients, differentiating CRC from precancerous or lesions [133], and a potential screening and predictive marker to immunotherapy [134]	The wide spread of the mutations over variable codons [134]	CBioportal, oncomine [132]

RAS mutation analysis	Excellent concordance with liver metastases in CRC patients who account for RAS mutations in exons 2 (codons 12 and 13), 3 (codons 59 and 61), and 4 (codons 117 and 146), predictive marker for monitoring the resistance to treatment with monoclonal antibodies (as monotherapy or combined with chemotherapy) [65]	Not declared	COSMIC, TCGA, GSEA, GO enrichment, KEGG, STRING, CPTAC, [125–127]

OncoBEAMTM RAS	One-step ultra-sensitive quantitative detection of plasma-derived KRAS and NRAS mutations for diagnosis, treatment, and immunotherapy monitoring of mCRC patients after surgery [84, 95], significant low threshold detection [67], kinetical assaying of the mutated haploid GE quantity [67], prognostic value of MAF indicator in mCRC patients [67]	Not declared	MPprimer, Ultiplex [135, 136]

IdyllaTM RAS	Accuracy sensitivity rate of ≤1% and ≤5 for KRAS mutations in exons 2, 3, and 4, respectively (potential assay for highly individualized anti-EGFR therapy and chemotherapy treatment decisions) [84]	Two-step assay of RAS mutational status with lower clinical sensitivity than OncoBEAM assay [84]	Biocartis Idylla™ System [84]

SSCP method	High sensitivity, specificity, for rapid diagnosis of hereditary nonpolyposis colorectal cancer (HNPCC) families based on hMLH1 and hMSH2 mutations with a good limit of detection (10%) [137–140]	The longest turnaround time [137–140]	GenBank, MUSCLE alignment program [141]

Direct sequencing	The gold standard, highly sensitive, and cost-effective, the quantitative measuring of an individual mutant allele in ctDNA for distinguishing CRC histological type [35, 142, 143]	Limited potency for low amount mutant sequences detection in full wild-type DNA sequence context [35, 142, 143]	SnackVar, SangeR [144, 145]

Long cell-free DNA fragment/*β*-globin ratio	Biomarker of early colorectal liver metastasis recurrence [146] (patient's monitoring after CRC surgery)	*β*-globin gene must also be analyzed in cfDNA as an index of overall impair [146]	Twoddpcr [147, 148], REST [149]

## Data Availability

The data used to support the findings of this study are included within the article.

## References

[1] Gonzalez-Pons M., Cruz-Correa M. (2020). Colorectal cancer disparities in latinos: genes vs. environment. *Advancing the Science of Cancer in Latinos*.

[2] Shi X., Duose D. Y., Mehrotra M. (2019). Non-invasive genotyping of metastatic colorectal cancer using circulating cell free DNA. *Cancer Genetics*.

[3] Naini M. A., Kavousipour S., Hasanzarini M., Nasrollah A., Monabati A., Mokarram P. (2018). O6-methyguanine-DNA methyl transferase (MGMT) promoter methylation in serum DNA of Iranian patients with colorectal cancer. *Asian Pacific Journal of Cancer Prevention*.

[4] Patel S. G., May F. P., Anderson J. C. (2022). Updates on age to start and stop colorectal cancer screening: recommendations from the U.S. multi-society task force on colorectal cancer. *Gastroenterology*.

[5] Libby G., Brewster D. H., McClements P. L. (2012). The impact of population-based faecal occult blood test screening on colorectal cancer mortality: a matched cohort study. *British Journal of Cancer*.

[6] Cheng Y.-W., Li Y.-C. (2021). Factors affecting the follow-up time after a positive result in the fecal occult blood test. *PLOS ONE*.

[7] Luo H., Shen K., Li B., Li R., Wang Z., Xie Z. (2020). Clinical significance and diagnostic value of serum NSE, CEA, CA19-9, CA125 and CA242 levels in colorectal cancer. *Oncology Letters*.

[8] Harlé A. (2020). Cell-free DNA in the management of colorectal cancer. *Tumor Liquid Biopsies*.

[9] Snyder M. W., Kircher M., Hill A. J., Daza R. M., Shendure J. (2016). Cell-free DNA comprises an in vivo nucleosome footprint that informs its tissues-of-origin. *Cell*.

[10] Liebs S., Keilholz U., Kehler I., Schweiger C., Haybäck J., Nonnenmacher A. (2019). Detection of mutations in circulating cell-free DNA in relation to disease stage in colorectal cancer. *Cancer Medicine*.

[11] Heider K., Wan J. C. M., Hall J. (2020). Detection of ctDNA from dried blood spots after DNA size selection. *Clinical Chemistry*.

[12] Sorber L., Zwaenepoel K., Jacobs J. (2019). Circulating cell-free DNA and RNA analysis as liquid biopsy: optimal centrifugation protocol. *Cancers*.

[13] Ju S., Chen C., Zhang J. (2022). Detection of circulating tumor cells: opportunities and challenges. *Biomarker Research*.

[14] Li X., Li Y., Shao W., Li Z., Zhao R., Ye Z. (2020). Strategies for enrichment of circulating tumor cells. *Translational Cancer Research*.

[15] Dianat-Moghadam H., Azizi M., Eslami-S Z. (2020). The role of circulating tumor cells in the metastatic cascade: biology, technical challenges, and clinical relevance. *Cancers*.

[16] Che J., Yu V., Garon E. B., Goldman J. W., Di Carlo D. (2017). Biophysical isolation and identification of circulating tumor cells. *Lab on a Chip*.

[17] Cheng Y.-H., Chen Y.-C., Lin E. (2019). Hydro-Seq enables contamination-free high-throughput single-cell RNA-sequencing for circulating tumor cells. *Nature Communications*.

[18] Hu X., Zang X., Lv Y. (2021). Detection of circulating tumor cells: advances and critical concerns. *Oncology Letters*.

[19] Deng Z., Wu S., Wang Y., Shi D. (2022). Circulating tumor cell isolation for cancer diagnosis and prognosis. *eBioMedicine*.

[20] Song Y., Tian T., Shi Y. (2017). Enrichment and single-cell analysis of circulating tumor cells. *Chemical Science*.

[21] Jie X.-X., Zhang X.-Y., Xu C.-J. (2017). Epithelial-to-mesenchymal transition, circulating tumor cells and cancer metastasis: mechanisms and clinical applications. *Oncotarget*.

[22] Descamps L., Le Roy D., Deman A.-L. (2022). Microfluidic-based technologies for CTC isolation: a review of 10 years of intense efforts towards liquid biopsy. *International Journal of Molecular Sciences*.

[23] Orrapin S., Thongkumkoon P., Udomruk S. (2023). Deciphering the biology of circulating tumor cells through single-cell RNA sequencing: implications for precision medicine in cancer. *International Journal of Molecular Sciences*.

[24] Dasari A., Morris V. K., Allegra C. J. (2020). ctDNA applications and integration in colorectal cancer: an NCI Colon and Rectal–Anal Task Forces whitepaper. *Nature Reviews Clinical Oncology*.

[25] Breitbach S., Tug S., Simon P. (2012). Circulating cell-free DNA: an up-coming molecular marker in exercise physiology. *Sports Medicine*.

[26] Hummel E. M., Hessas E., Müller S. (2018). Cell-free DNA release under psychosocial and physical stress conditions. *Translational Psychiatry*.

[27] Mauger F., Dulary C., Daviaud C., Deleuze J.-F., Tost J. (2015). Comprehensive evaluation of methods to isolate, quantify, and characterize circulating cell-free DNA from small volumes of plasma. *Analytical and Bioanalytical Chemistry*.

[28] Ammerlaan W., Betsou F. (2019). Biospecimen science of blood for cfDNA genetic analyses. *Current Pathobiology Reports*.

[29] Page K., Guttery D. S., Zahra N. (2013). Influence of plasma processing on recovery and analysis of circulating nucleic acids. *PLOS ONE*.

[30] Diefenbach R. J., Jenny H. L., Kefford R. F., Rizos H. (2018). Evaluation of commercial kits for purification of circulating free DNA. *Cancer Genetics*.

[31] Suraj S., Dhar C., Srivastava S. (2017). Circulating nucleic acids: an analysis of their occurrence in malignancies. *Biomedical Reports*.

[32] Gao Q., Zeng Q., Wang Z. (2022). Circulating cell-free DNA for cancer early detection. *The Innovation*.

[33] Vymetalkova V., Cervena K., Bartu L., Vodicka P. (2018). Circulating cell-free DNA and colorectal cancer: a systematic review. *International Journal of Molecular Sciences*.

[34] Jiang N., Pan J., Fang S. (2019). Liquid biopsy: circulating exosomal long noncoding RNAs in cancer. *Clinica Chimica Acta*.

[35] Tóth K., Patai Á. V., Kalmár A. (2017). Circadian rhythm of methylated septin 9, cell-free DNA amount and tumor markers in colorectal cancer patients. *Pathology & Oncology Research*.

[36] de la Cruz F. F., Corcoran R. B. (2018). Methylation in cell-free DNA for early cancer detection. *Annals of Oncology*.

[37] Huang C.-C., Du M., Wang L. (2019). Bioinformatics analysis for circulating cell-free DNA in cancer. *Cancers*.

[38] Ahlquist D. A., Skoletsky J. E., Boynton K. A. (2000). Colorectal cancer screening by detection of altered human DNA in stool: feasibility of a multitarget assay panel. *Gastroenterology*.

[39] Olmedillas-López S., Lévano-Linares D. C., Alexandre C. L. A. (2017). Detection of *KRAS* G12D in colorectal cancer stool by droplet digital PCR. *World Journal of Gastroenterology*.

[40] Onouchi S., Matsushita H., Moriya Y. (2008). New method for colorectal cancer diagnosis based on SSCP analysis of DNA from exfoliated colonocytes in naturally evacuated feces. *Anticancer Research*.

[41] Smith T., Heger A., Sudbery I. (2017). UMI-tools: modeling sequencing errors in unique molecular identifiers to improve quantification accuracy. *Genome Research*.

[42] Fang Q., Yuan Z., Hu H., Zhang W., Wang G., Wang X. (2023). Genome-wide discovery of circulating cell-free DNA methylation biomarkers for colorectal cancer detection. *Clinical Epigenetics*.

[43] Olova N., Krueger F., Andrews S. (2018). Comparison of whole-genome bisulfite sequencing library preparation strategies identifies sources of biases affecting DNA methylation data. *Genome Biology*.

[44] Grehl C., Kuhlmann M., Becker C., Glaser B., Grosse I. (2018). How to design a whole-genome bisulfite sequencing experiment. *Epigenomes*.

[45] Kim H., Sim M., Park N., Kwon K., Kim J., Kim J. (2022). msPIPE: a pipeline for the analysis and visualization of whole-genome bisulfite sequencing data. *BMC Bioinformatics*.

[46] Wöste M., Leitão E., Laurentino S., Horsthemke B., Rahmann S., Schröder C. (2020). wg-blimp: an end-to-end analysis pipeline for whole genome bisulfite sequencing data. *BMC Bioinformatics*.

[47] Liao W.-W., Yen M.-R., Ju E., Hsu F.-M., Lam L., Chen P.-Y. (2015). MethGo: a comprehensive tool for analyzing whole-genome bisulfite sequencing data. *BMC Genomics*.

[48] Pillman K. A., Goodall G. J., Bracken C. P., Gantier M. P. (2019). miRNA length variation during macrophage stimulation confounds the interpretation of results: implications for miRNA quantification by RT-qPCR. *RNA*.

[49] Mestdagh P., Hartmann N., Baeriswyl L. (2014). Evaluation of quantitative miRNA expression platforms in the microRNA quality control (miRQC) study. *Nature Methods*.

[50] Biosystems A (2015). *Real-Time PCR Handbook*.

[51] Smolander J., Khan S., Singaravelu K. (2021). Evaluation of tools for identifying large copy number variations from ultra-low-coverage whole-genome sequencing data. *BMC Genomics*.

[52] Raman L., Dheedene A., De Smet M., Van Dorpe J., Menten B. (2019). WisecondorX: improved copy number detection for routine shallow whole-genome sequencing. *Nucleic Acids Research*.

[53] Xi R., Lee S., Xia Y., Kim T.-M., Park P. J. (2016). Copy number analysis of whole-genome data using BIC-seq2 and its application to detection of cancer susceptibility variants. *Nucleic Acids Research*.

[54] Hrdlickova R., Toloue M., Tian B. (2017). RNA-Seq methods for transcriptome analysis. *WIREs RNA*.

[55] Whitley S. K., Horne W. T., Kolls J. K. (2016). Research techniques made simple: methodology and clinical applications of RNA sequencing. *Journal of Investigative Dermatology*.

[56] Ding Y., Li J., Li T., Deng X. (2023). Protocol for integrated analysis of bacterial RNA-seq and ChIP-seq data for establishing a co-expression network. *STAR Protocols*.

[57] Koeppel F., Blanchard S., Jovelet C. (2017). Whole exome sequencing for determination of tumor mutation load in liquid biopsy from advanced cancer patients. *PLOS ONE*.

[58] Ono Y., Sugitani A., Karasaki H. (2017). An improved digital polymerase chain reaction protocol to capture low-copy *KRAS* mutations in plasma cell-free DNA by resolving ‘subsampling’ issues. *Molecular Oncology*.

[59] van der Werf I. M., Kooy R. F., Vandeweyer G. (2015). A robust protocol to increase NimbleGen SeqCap EZ multiplexing capacity to 96 samples. *PLOS ONE*.

[60] Kuypers J., Jerome K. R. (2017). Applications of digital PCR for clinical microbiology. *Journal of Clinical Microbiology*.

[61] Campomenosi P., Gini E., Noonan D. M. (2016). A comparison between quantitative PCR and droplet digital PCR technologies for circulating microRNA quantification in human lung cancer. *BMC Biotechnology*.

[62] Olmedillas-López S., García-Olmo D. C., García-Arranz M., Peiró-Pastor R., Aguado B., García-Olmo D. (2018). Liquid biopsy by NGS: differential presence of exons (DPE) in cell-free DNA reveals different patterns in metastatic and nonmetastatic colorectal cancer. *Cancer Medicine*.

[63] Porter A., Natsuhara M., Daniels G. A. (2020). Next generation sequencing of cell free circulating tumor DNA in blood samples of recurrent and metastatic head and neck cancer patients. *Translational Cancer Research*.

[64] Liu S., Wang J. (2022). Current and future perspectives of cell-free DNA in liquid biopsy. *Current Issues in Molecular Biology*.

[65] Harle A., Gavoille C., Bouche O. (2019). cfDNA for accurate determination of RAS and BRAF mutations using OncoBEAM liquid biopsy in metastatic colorectal cancer patients: results of the real-world multicentric ColoBEAM study. *Journal of Clinical Oncology*.

[66] Nykel A., Kaszkowiak M., Fendler W., Gach A. (2019). Chip-based digital PCR approach provides a sensitive and cost-effective single-day screening tool for common fetal aneuploidies—a proof of concept study. *International Journal of Molecular Sciences*.

[67] Garcia J., Forestier J., Dusserre E. (2018). Cross-platform comparison for the detection of RAS mutations in cfDNA (ddPCR biorad detection assay, BEAMing assay, and NGS strategy). *Oncotarget*.

[68] Janku F., Huang H. J., Fujii T. (2017). MultiplexKRASG12/G13 mutation testing of unamplified cell-free DNA from the plasma of patients with advanced cancers using droplet digital polymerase chain reaction. *Annals of Oncology*.

[69] Orue A., Rieber M., Wang H. (2016). Optimized multiplex detection of 7 KRAS mutations by Taqman Allele-specific qPCR. *PLOS ONE*.

[70] Nakajima H., Kotani D., Bando H. (2021). REMARRY and PURSUIT trials: liquid biopsy-guided rechallenge with anti-epidermal growth factor receptor (EGFR) therapy with panitumumab plus irinotecan for patients with plasma RAS wild-type metastatic colorectal cancer. *BMC Cancer*.

[71] Khandelwal A. R., Greer A. H., Hamiter M. (2020). Comparing cell-free circulating tumor DNA mutational profiles of disease-free and nonresponders patients with oropharyngeal squamous cell carcinoma. *Laryngoscope Investigative Otolaryngology*.

[72] Takeda K., Yamada T., Takahashi G. (2019). Analysis of colorectal cancer-related mutations by liquid biopsy: utility of circulating cell-free DNA and circulating tumor cells. *Cancer Science*.

[73] Conte D., Verri C., Borzi C. (2015). Novel method to detect microRNAs using chip-based quantStudio 3D digital PCR. *BMC Genomics*.

[74] Pervez M. T., Hasnain M. J., Abbas S. H., Moustafa M. F., Aslam N., Shah S. S. M. (2022). A comprehensive review of performance of next-generation sequencing platforms. *BioMed Research International*.

[75] Kang J.-K., Heo S., Kim H.-P. (2020). Liquid biopsy-based tumor profiling for metastatic colorectal cancer patients with ultra-deep targeted sequencing. *PLOS ONE*.

[76] Esfandyarpour H., Parizi K. B., Barmi M. R. (2019). High accuracy DNA sequencing on a small, scalable platform via electrical detection of single base incorporations.

[77] Pearman W. S., Freed N. E., Silander O. K. (2020). Testing the advantages and disadvantages of short- and long-read eukaryotic metagenomics using simulated reads. *BMC Bioinformatics*.

[78] Fadda A., Gentilini D., Moi L. (2018). Colorectal cancer early methylation alterations affect the crosstalk between cell and surrounding environment, tracing a biomarker signature specific for this tumor. *International Journal of Cancer*.

[79] Masters A., Harrison P. (2013). Platelet counting with the BD Accuri^TM^ C6 flow cytometer. *Platelets*.

[80] Yamada T., Iwai T., Takahashi G. (2016). Utility of *KRAS* mutation detection using circulating cell-free DNA from patients with colorectal cancer. *Cancer Science*.

[81] Olivier M. (2005). The Invader® assay for SNP genotyping. *Mutation Research/Fundamental and Molecular Mechanisms of Mutagenesis*.

[82] Allawi H. T., Li H., Sander T. (2006). Invader plus method detects herpes simplex virus in cerebrospinal fluid and simultaneously differentiates types 1 and 2. *Journal of Clinical Microbiology*.

[83] Liskova A., Samec M., Koklesova L., Giordano F. A., Kubatka P., Golubnitschaja O. (2020). Liquid biopsy is instrumental for 3PM dimensional solutions in cancer management. *Journal of Clinical Medicine*.

[84] Vivancos A., Aranda E., Benavides M. (2019). Comparison of the clinical sensitivity of the Idylla platform and the OncoBEAM RAS CRC assay for KRAS mutation detection in liquid biopsy samples. *Scientific Reports*.

[85] Picardo F., Romanelli A., Muinelo-Romay L. (2019). Diagnostic and prognostic value of B4GALT1 hypermethylation and its clinical significance as a novel circulating cell-free DNA biomarker in colorectal cancer. *Cancers*.

[86] Zhao G., Li H., Yang Z. (2019). Multiplex methylated DNA testing in plasma with high sensitivity and specificity for colorectal cancer screening. *Cancer Medicine*.

[87] Chen Y., Wang Z., Zhao G. (2019). Performance of a novel blood-based early colorectal cancer screening assay in remaining serum after the blood biochemical test. *Disease Markers*.

[88] Bronkhorst A. J., Ungerer V., Holdenrieder S. (2019). The emerging role of cell-free DNA as a molecular marker for cancer management. *Biomolecular Detection and Quantification*.

[89] Meddeb R., Dache Z. A. A., Thezenas S. (2019). Quantifying circulating cell-free DNA in humans. *Scientific Reports*.

[90] Jiang F., Yang X., He X., Yang M. (2019). Circulating DNA, a potentially sensitive and specific diagnostic tool for future medicine. *Dose-Response*.

[91] Ding Y., Li W., Wang K., Xu C., Hao M., Ding L. (2020). Perspectives of the application of liquid biopsy in colorectal cancer. *BioMed Research International*.

[92] Parikh A. R., Leshchiner I., Elagina L. (2019). Liquid versus tissue biopsy for detecting acquired resistance and tumor heterogeneity in gastrointestinal cancers. *Nature Medicine*.

[93] Day E., Dear P. H., McCaughan F. (2013). Digital PCR strategies in the development and analysis of molecular biomarkers for personalized medicine. *Methods*.

[94] Olmedillas-López S., García-Arranz M., García-Olmo D. (2017). Current and emerging applications of droplet digital PCR in oncology. *Molecular Diagnosis & Therapy*.

[95] Franczak C., Witz A., Geoffroy K. (2020). Evaluation of KRAS, NRAS and BRAF mutations detection in plasma using an automated system for patients with metastatic colorectal cancer. *PLOS ONE*.

[96] Bando H., Kagawa Y., Kato T. (2019). A multicentre, prospective study of plasma circulating tumour DNA test for detecting RAS mutation in patients with metastatic colorectal cancer. *British Journal of Cancer*.

[97] Hao T. B., Shi W., Shen X. J. (2014). Circulating cell-free DNA in serum as a biomarker for diagnosis and prognostic prediction of colorectal cancer. *British Journal of Cancer*.

[98] Bronkhorst A. J., Ungerer V., Oberhofer A. (2022). New perspectives on the importance of cell-free DNA biology. *Diagnostics*.

[99] Sato A., Nakashima C., Abe T. (2018). Investigation of appropriate pre-analytical procedure for circulating free DNA from liquid biopsy. *Oncotarget*.

[100] Warton K., Lin V., Navin T. (2014). Methylation-capture and next-generation sequencing of free circulating DNA from human plasma. *BMC Genomics*.

[101] Parpart-Li S., Bartlett B., Popoli M. (2017). The effect of preservative and temperature on the analysis of circulating tumor DNA. *Clinical Cancer Research*.

[102] Holmes E. E., Jung M., Meller S. (2014). Performance evaluation of kits for bisulfite-conversion of DNA from tissues, cell lines, FFPE tissues, aspirates, lavages, effusions, plasma, serum, and urine. *PLOS ONE*.

[103] Polatoglou E., Mayer Z., Ungerer V., Bronkhorst A. J., Holdenrieder S. (2022). Isolation and quantification of plasma cell-free DNA using different manual and automated methods. *Diagnostics*.

[104] Pérez-Barrios C., Nieto-Alcolado I., Torrente M. (2016). Comparison of methods for circulating cell-free DNA isolation using blood from cancer patients: impact on biomarker testing. *Translational Lung Cancer Research*.

[105] Keeley B., Stark A., Pisanic T. R. (2013). Extraction and processing of circulating DNA from large sample volumes using methylation on beads for the detection of rare epigenetic events. *Clinica Chimica Acta*.

[106] Norton S. E., Lechner J. M., Williams T., Fernando M. R. (2013). A stabilizing reagent prevents cell-free DNA contamination by cellular DNA in plasma during blood sample storage and shipping as determined by digital PCR. *Clinical Biochemistry*.

[107] Çayir A., Coskun M., Coskun M., Cobanoglu H. (2018). DNA damage and circulating cell free DNA in greenhouse workers exposed to pesticides. *Environmental and Molecular Mutagenesis*.

[108] Elshimali Y., Khaddour H., Sarkissyan M., Wu Y., Vadgama J. (2013). The clinical utilization of circulating cell free DNA (CCFDNA) in blood of cancer patients. *International Journal of Molecular Sciences*.

[109] Keshavarz Z., Moezzi L., Ranjbaran R. (2015). Evaluation of a modified DNA extraction method for isolation of cell-free fetal DNA from maternal serum. *Avicenna Journal of Medical Biotechnology*.

[110] Hufnagl C., Stöcher M., Moik M., Geisberger R., Greil R. (2013). A modified phenol-chloroform extraction method for isolating circulating cell free DNA of tumor patients. *Journal of Nucleic Acids Investigation*.

[111] Maxwell E. Maxwell® 16 DNA purification kits.

[112] Armand-Labit V., Pradines A. (2017). Circulating cell-free microRNAs as clinical cancer biomarkers. *Biomolecular Concepts*.

[113] Xia S., Huang C.-C., Le M. (2015). Genomic variations in plasma cell free DNA differentiate early stage lung cancers from normal controls. *Lung Cancer*.

[114] Hovelson D. H., Liu C.-J., Wang Y. (2017). Rapid, ultra low coverage copy number profiling of cell-free DNA as a precision oncology screening strategy. *Oncotarget*.

[115] Shi L., Tang X., Qian M. (2018). A SIRT1-centered circuitry regulates breast cancer stemness and metastasis. *Oncogene*.

[116] Shiovitz S., Grady W. M. (2015). Molecular markers predictive of chemotherapy response in colorectal cancer. *Current Gastroenterology Reports*.

[117] Fu Y., Ye Y., Liu X. (2021). Analyzing microsatellite instability and gene mutation in circulating cell-free DNA to monitor colorectal cancer progression. *Translational Cancer Research*.

[118] Hallermayr A., Wohlfrom T., Steinke-Lange V. (2022). Somatic copy number alteration and fragmentation analysis in circulating tumor DNA for cancer screening and treatment monitoring in colorectal cancer patients. *Journal of Hematology & Oncology*.

[119] Siraj A. K., Bu R., Masoodi T. (2022). Exome sequencing revealed comparable frequencies of RNF43 and BRAF mutations in Middle Eastern colorectal cancer. *Scientific Reports*.

[120] Cai Z., Wang Z., Liu C. (2020). Detection of microsatellite instability from circulating tumor DNA by targeted deep sequencing. *Journal of Molecular Diagnostics*.

[121] Normanno N., Cervantes A., Ciardiello F., De Luca A., Pinto C. (2018). The liquid biopsy in the management of colorectal cancer patients: current applications and future scenarios. *Cancer Treatment Reviews*.

[122] Pabinger S., Thallinger G. G., Snajder R., Eichhorn H., Rader R., Trajanoski Z. (2009). QPCR: application for real-time PCR data management and analysis. *BMC Bioinformatics*.

[123] Xu X.-M., Qian J.-C., Cai Z. (2012). DNA alterations of microsatellite DNA, p53, APC and K-ras in Chinese colorectal cancer patients. *European Journal of Clinical Investigation*.

[124] Yu H., Han L., Yuan J., Sun Y. (2020). Circulating tumor cell free DNA from plasma and urine in the clinical management of colorectal cancer. *Cancer Biomarkers*.

[125] Roy A., Deb M., Niharika, Parbin S., Shilpi A., Patra S. K. (2022). Comprehensive bioinformatic analyses of KRAS mutations and deciphering chromatin modification landscape of Caveolin-1 gene by lipid raft destabilization induced modulation of RAS-MAPK axis in colon cancer. *Advances in Cancer Biology-Metastasis*.

[126] Saliani M., Jalal R., Javadmanesh A. (2022). Differential expression analysis of genes and long non-coding RNAs associated with KRAS mutation in colorectal cancer cells. *Scientific Reports*.

[127] Lam K. K., Tang C. L., Tan E., Wong S. H., Cheah P. Y. (2022). *KRAS* mutation-independent downregulation of MAPK/PI3K signaling in colorectal cancer. *Molecular Oncology*.

[128] Pereira R., Oliveira J., Sousa M. (2020). Bioinformatics and computational tools for next-generation sequencing analysis in clinical genetics. *Journal of Clinical Medicine*.

[129] Sanz-Garcia E., Argiles G., Elez E., Tabernero J. (2017). BRAF mutant colorectal cancer: prognosis, treatment, and new perspectives. *Annals of Oncology*.

[130] Luo H., Zhao Q., Wei W. (2020). Circulating tumor DNA methylation profiles enable early diagnosis, prognosis prediction, and screening for colorectal cancer. *Science Translational Medicine*.

[131] Lee J. J., Chu E. (2014). The role of predictive molecular biomarkers for the treatment of metastatic colorectal cancer. *Current Colorectal Cancer Reports*.

[132] van den Driest L., Johnson C. H., Rattray N. J. W., Rattray Z. (2021). Development of an accessible gene expression bioinformatics pipeline to study driver mutations of colorectal cancer. *Alternatives to Laboratory Animals*.

[133] Aitchison A., Hakkaart C., Day R. C., Morrin H. R., Frizelle F. A., Keenan J. I. (2020). APC mutations are not confined to hotspot regions in early-onset colorectal cancer. *Cancers*.

[134] Diehl F., Li M., Dressman D. (2005). Detection and quantification of mutations in the plasma of patients with colorectal tumors. *Proceedings of the National Academy of Sciences*.

[135] Shen Z., Qu W., Wang W. (2010). MPprimer: a program for reliable multiplex PCR primer design. *BMC Bioinformatics*.

[136] Yuan J., Yi J., Zhan M. (2021). The web-based multiplex PCR primer design software ultiplex and the associated experimental workflow: up to 100-plex multiplicity. *BMC Genomics*.

[137] Er T.-K., Chang Y.-S., Yeh K.-T., Chang T.-J., Chang J.-G. (2010). Comparison of two different screening methods for the KRAS mutation in colorectal cancer. *Clinical Laboratory*.

[138] Lewandowska M. A., Jóźwicki W., Żurawski B. (2013). KRAS and BRAF mutation analysis in colorectal adenocarcinoma specimens with a low percentage of tumor cells. *Molecular Diagnosis & Therapy*.

[139] Kumasaka A., Matsumoto N., Mukae S. (2016). Rapid and specific screening assay for KRAS oncogene mutation by a novel gene amplification method. *Anticancer Research*.

[140] Al-Haggar M. (2013). Evolving molecular methods for detection of mutations. *Gene Technology*.

[141] Pasookhush P., Usmani A., Suwannahong K. (2021). Single-strand conformation polymorphism fingerprint method for dictyostelids. *Frontiers in Microbiology*.

[142] Jancik S., Drabek J., Berkovcova J. (2012). A comparison of direct sequencing, pyrosequencing, high resolution melting analysis, TheraScreen DxS, and the K-ras StripAssay for detecting KRAS mutations in non small cell lung carcinomas. *Journal of Experimental & Clinical Cancer Research*.

[143] Oh H.-S., Kwon H., Park S. (2018). Comparison of immunohistochemistry and direct sanger sequencing for detection of the *BRAF*^V600E^ mutation in thyroid neoplasm. *Endocrinology and Metabolism*.

[144] Schmid K., Dohmen H., Ritschel N. (2022). SangeR: the high-throughput sanger sequencing analysis pipeline. *Bioinformatics Advances*.

[145] Kim Y.-G., Kim M. J., Lee J.-S. (2021). SnackVar: an open-source software for sanger sequencing analysis optimized for clinical use. *The Journal of Molecular Diagnostics*.

[146] Iwai T., Yamada T., Takahashi G. (2020). Circulating cell-free long DNA fragments predict post-hepatectomy recurrence of colorectal liver metastases. *European Journal of Surgical Oncology*.

[147] Chiu A., Ayub M., Dive C., Brady G., Miller C. J. (2017). twoddpcr: an R/Bioconductor package and shiny app for droplet digital PCR analysis. *Bioinformatics*.

[148] Koval A. P., Khromova A. S., Blagodatskikh K. A. (2023). Application of PCR-based approaches for evaluation of cell-free DNA fragmentation in colorectal cancer. *Frontiers in Molecular Biosciences*.

[149] Garrido-Navas M. C., García-Díaz A., Molina-Vallejo M. P. (2020). The polemic diagnostic role of tp53 mutations in liquid biopsies from breast, colon and lung cancers. *Cancers*.

[150] Wang J.-Y., Hsieh J.-S., Chang M.-Y. (2004). Molecular detection of *APC*, K- *ras*, and *p53* mutations in the serum of colorectal cancer patients as circulating biomarkers. *World Journal of Surgery*.

[151] Hong S. N. (2018). Genetic and epigenetic alterations of colorectal cancer. *Intestinal Research*.

[152] Boland C. R., Goel A. (2010). Microsatellite instability in colorectal cancer. *Gastroenterology*.

[153] Sun Q., Pastor L., Du J. (2021). A novel xenonucleic acid-mediated molecular clamping technology for early colorectal cancer screening. *PLOS ONE*.

[154] Shen C. X., Hu L. H., Xia L., Li Y. R. (2008). Quantitative real-time RT-PCR detection for survivin, CK20 and CEA in peripheral blood of colorectal cancer patients. *Japanese Journal of Clinical Oncology*.

[155] Vaiopoulos A. G., Kostakis I. D., Gkioka E. (2014). Detection of circulating tumor cells in colorectal and gastric cancer using a multiplex PCR assay. *Anticancer Research*.

[156] Bertorelle R., Rampazzo E., Pucciarelli S., Nitti D., De Rossi A. (2014). Telomeres, telomerase and colorectal cancer. *World Journal of Gastroenterology*.

[157] Singh N., Rashid S., Rashid S., Dash N. R., Gupta S., Saraya A. (2020). Clinical significance of promoter methylation status of tumor suppressor genes in circulating DNA of pancreatic cancer patients. *Journal of Cancer Research and Clinical Oncology*.

[158] Jeepalyam S., Sheel A., Ejaz A., Miller E., Manne A. (2023). Is cell-free DNA testing in hepatocellular carcinoma ready for prime time?. *International Journal of Molecular Sciences*.

[159] Tan S.-H., Ida H., Lau Q.-C. (2007). Detection of promoter hypermethylation in serum samples of cancer patients by methylation-specific polymerase chain reaction for tumour suppressor genes including RUNX3. *Oncology Reports*.

[160] Aghagolzadeh P., Radpour R. (2016). New trends in molecular and cellular biomarker discovery for colorectal cancer. *World Journal of Gastroenterology*.

[161] Wallner M., Herbst A., Behrens A. (2006). Methylation of serum DNA is an independent prognostic marker in colorectal cancer. *Clinical Cancer Research*.

[162] Yang C., Zhang Y., Xu X., Li W. (2019). Molecular subtypes based on DNA methylation predict prognosis in colon adenocarcinoma patients. *Aging*.

[163] Wang X., Kuang Y.-Y., Hu X.-T. (2014). Advances in epigenetic biomarker research in colorectal cancer. *World Journal of Gastroenterology*.

[164] Bi F., Wang Q., Dong Q., Wang Y., Zhang L., Zhang J. (2020). Circulating tumor DNA in colorectal cancer: opportunities and challenges. *American Journal of Translational Research*.

[165] Ladabaum U., Alvarez-Osorio L., Rösch T., Brueggenjuergen B. (2014). Cost-effectiveness of colorectal cancer screening in Germany: current endoscopic and fecal testing strategies versus plasma methylated Septin 9 DNA. *Endoscopy International Open*.

[166] Church T. R., Wandell M., Lofton-Day C. (2014). Prospective evaluation of methylated *SEPT9* in plasma for detection of asymptomatic colorectal cancer. *Gut*.

[167] To K. K. W., Tong C. W. S., Wu M., Cho W. C. S. (2018). MicroRNAs in the prognosis and therapy of colorectal cancer: from bench to bedside. *World Journal of Gastroenterology*.

[168] Bhaskaran M., Mohan M. (2014). MicroRNAs: history, biogenesis, and their evolving role in animal development and disease. *Veterinary Pathology*.

[169] Niveditha D., Jasoria M., Narayan J. (2020). Common and unique microRNAs in multiple carcinomas regulate similar network of pathways to mediate cancer progression. *Scientific Reports*.

[170] Ahmed F. E., Ahmed N. C., Vos P. W. (2013). Diagnostic microRNA markers to screen for sporadic human colon cancer in stool: I. proof of principle. *Cancer Genomics & Proteomics*.

[171] Giráldez M. D., Lozano J. J., Ramírez G. (2013). Circulating microRNAs as biomarkers of colorectal cancer: results from a genome-wide profiling and validation study. *Clinical Gastroenterology and Hepatology*.

[172] Kanaan Z., Roberts H., Eichenberger M. R. (2013). A plasma microRNA panel for detection of colorectal adenomas. *Annals of Surgery*.

[173] Ahmed F. E., Amed N. C., Vos P. W. (2012). Diagnostic microRNA markers to screen for sporadic human colon cancer in blood. *Cancer Genomics & Proteomics*.

[174] Huang Z., Huang D., Ni S., Peng Z., Sheng W., Du X. (2010). Plasma microRNAs are promising novel biomarkers for early detection of colorectal cancer. *International Journal of Cancer*.

[175] Pu X.-X., Huang G.-L., Guo H.-Q. (2010). Circulating miR-221 directly amplified from plasma is a potential diagnostic and prognostic marker of colorectal cancer and is correlated with p53 expression. *Journal of Gastroenterology and Hepatology*.

[176] Carter J. V., Galbraith N. J., Yang D., Burton J. F., Walker S. P., Galandiuk S. (2017). Blood-based microRNAs as biomarkers for the diagnosis of colorectal cancer: a systematic review and meta-analysis. *British Journal of Cancer*.

[177] Ganepola G. A. P., Nizin J., Rutledge J. R., Chang D. H. (2014). Use of blood-based biomarkers for early diagnosis and surveillance of colorectal cancer. *World Journal of Gastrointestinal Oncology*.

[178] Arita T., Ichikawa D., Konishi H. (2013). Circulating long non-coding RNAs in plasma of patients with gastric cancer. *Anticancer Research*.

[179] Dong L., Qi P., Xu M.-D. (2015). Circulating CUDR, LSINCT-5 and PTENP1 long noncoding RNAs in sera distinguish patients with gastric cancer from healthy controls. *International Journal of Cancer*.

[180] Tong Y.-S., Wang X.-W., Zhou X.-L. (2015). Identification of the long non-coding RNA *POU3F3* in plasma as a novel biomarker for diagnosis of esophageal squamous cell carcinoma. *Molecular Cancer*.

[181] Graham L. D., Pedersen S. K., Brown G. S. (2011). Colorectal neoplasia differentially expressed (CRNDE), a novel gene with elevated expression in colorectal adenomas and adenocarcinomas. *Genes & Cancer*.

[182] Zhao W., Song M., Zhang J., Kuerban M., Wang H. (2015). Combined identification of long non-coding RNA CCAT1 and HOTAIR in serum as an effective screening for colorectal carcinoma. *International Journal of Clinical and Experimental Pathology*.

[183] Malla M., Loree J. M., Kasi P. M., Parikh A. R. (2022). Using circulating tumor DNA in colorectal cancer: current and evolving practices. *Journal of Clinical Oncology*.

[184] Marcuello M., Vymetalkova V., Neves R. P. L. (2019). Circulating biomarkers for early detection and clinical management of colorectal cancer. *Molecular Aspects of Medicine*.

[185] Wang Z., Sun K., Jing C., Cao H., Ma R., Wu J. (2019). Comparison of droplet digital PCR and direct Sanger sequencing for the detection of the *BRAF*^V600E^ mutation in papillary thyroid carcinoma. *Journal of Clinical Laboratory Analysis*.

[186] Colmenares R., Álvarez N., Barrio S., Martínez-López J., Ayala R. (2022). The minimal residual disease using liquid biopsies in hematological malignancies. *Cancers*.

[187] Moding E. J., Nabet B. Y., Alizadeh A. A., Diehn M. (2021). Detecting liquid remnants of solid tumors: circulating tumor DNA minimal residual disease. *Cancer Discovery*.

[188] McDonald B. R., Contente-Cuomo T., Sammut S.-J. (2019). Personalized circulating tumor DNA analysis to detect residual disease after neoadjuvant therapy in breast cancer. *Science Translational Medicine*.

[189] Peng Y., Mei W., Ma K., Zeng C. (2021). Circulating tumor DNA and minimal residual disease (MRD) in solid tumors: current horizons and future perspectives. *Frontiers in Oncology*.

[190] Parikh A. R., Van Seventer E. E., Siravegna G. (2021). Minimal residual disease detection using a plasma-only circulating tumor DNA assay in patients with colorectal cancer. *Clinical Cancer Research*.

[191] Chen G., Peng J., Xiao Q. (2021). Postoperative circulating tumor DNA as markers of recurrence risk in stages II to III colorectal cancer. *Journal of Hematology & Oncology*.

[192] Kasi A., Abbasi S., Handa S. (2020). Total neoadjuvant therapy vs standard therapy in locally advanced rectal cancer: a systematic review and meta-analysis. *JAMA Network Open*.

[193] Murahashi S., Akiyoshi T., Sano T. (2020). Serial circulating tumour DNA analysis for locally advanced rectal cancer treated with preoperative therapy: prediction of pathological response and postoperative recurrence. *British Journal of Cancer*.

[194] Rampazzo E., Del Bianco P., Bertorelle R. (2018). The predictive and prognostic potential of plasma telomerase reverse transcriptase (TERT) RNA in rectal cancer patients. *British Journal of Cancer*.

